# Compactness Aromaticity of Atoms in Molecules

**DOI:** 10.3390/ijms11041269

**Published:** 2010-03-26

**Authors:** Mihai V. Putz

**Affiliations:** 1 Laboratory of Computational and Structural Physical Chemistry, Chemistry Department, West University of Timişoara, Pestalozzi Street No.16, Timişoara, RO-300115, Romania; E-Mail: mvputz@cbg.uvt.ro or mv_putz@yahoo.com; Tel.: +40-0256-592-633; Fax: +40-0256-592-620; 2 “Nicolas Georgescu-Roegen” Forming and Researching Center of West University of Timişoara, 4th, Oituz Street, Timişoara, RO-300086, Romania

**Keywords:** chemical reactivity principles, polarizability, electronegativity, chemical hardness, quantum semi-empirical methods, quantum *ab initio* methods, aromaticity rules

## Abstract

A new aromaticity definition is advanced as the compactness formulation through the ratio between atoms-in-molecule and orbital molecular facets of the same chemical reactivity property around the pre- and post-bonding stabilization limit, respectively. Geometrical reactivity index of polarizability was assumed as providing the benchmark aromaticity scale, since due to its observable character; with this occasion new Hydrogenic polarizability quantum formula that recovers the exact value of 4.5 *a*_0_^3^ for Hydrogen is provided, where *a*_0_ is the Bohr radius; a polarizability based–aromaticity scale enables the introduction of five referential aromatic rules (Aroma 1 to 5 Rules). With the help of these aromatic rules, the aromaticity scales based on energetic reactivity indices of electronegativity and chemical hardness were computed and analyzed within the major semi-empirical and *ab initio* quantum chemical methods. Results show that chemical hardness based-aromaticity is in better agreement with polarizability based-aromaticity than the electronegativity-based aromaticity scale, while the most favorable computational environment appears to be the quantum semi-empirical for the first and quantum *ab initio* for the last of them, respectively.

## 1. Introduction

In conceptual chemistry, the aromaticity notion stands as one of the main pillars of structural understanding of molecular stability and reactivity; it spans all relevant periods of modern chemistry, suffering continuous revivals as the development of the physico-mathematical methods allows [1–5].

Historically, the custom definition of aromaticity is that it characterizes the planar molecules with (4*n* + 2) *π*-electrons [6], favoring substitution while resisting addition reactions [7,8], corresponds with high stability respecting the anti-aromatic structure [9], and ultimately correlates with low diamagnetism or magnetic susceptibility [10,11]. However, through the discovery of the non-planar feature of the most aromatic structure—benzene [12], along the generalizations of the Hückel rule for conjugated systems leading with the conjugated circuits [13,14], topological conjugated structures [15–17], up to the aromatic zones of molecular fragments [18,19], the aromaticity concept appears as a quite versatile concept that still needs proper quantification.

The main difficulty in capping the observability character of aromaticity may reside in the fact it does not directly relate to the ground state energy of molecules, but rather with their excited and valence orbitals–a condition closely related with the *π* electrons delocalized in the structure. Such *aromaticity paradox* of quantifying molecular stability (that usually is conducted by a Variational algorithm towards the *ground state*) by means of *frontier pi electrons*, may be considered as the main origin for the most inconsistencies and controversial points relating to the aromaticity concept in general and of its scales’ realization in particular.

Still, advancing aromaticity scales that employ certain physico-chemical molecular properties are useful at least for understanding whether certain molecular properties are inter-related and whether they enter or not the aromaticity sphere of definition. For that, the aromaticity scales should be always judged and validated in a comparative manner through concluding merely qualitatively upon the benchmarking degree of inter-correlation. This way, the aromaticity concept works best within the “transitivity thinking”: if the scale *A*_1_ correlates with the scales *A*_2_ and *A*_3_ with the same degree then the properties underlying the last two scales should be as well correlated and they may be regarded as different faces of the same molecular property. In short, the aromaticity concept and especially through its scales has the role of ordering among the molecular properties in general and of reactivity indices in special.

Returning to the aromaticity definition, it seems that the two main routes for its quantitative evaluation are the *energetic* and *geometric* ways; although within a Variational approach they should be closely related, *i.e.*, minimum global energy corresponds with the geometry optimization, since the aromaticity paradox described above the two sides of molecular structures open two different approaches for introducing quantitative indices of aromaticity. For instance, based on geometric (also extended to topological) criteria, the consecrated harmonic oscillator based-molecular aromaticity (HOMA) [20,21] and the recent topological paths and aromatic zones (TOPAZ) [18], as well as the ultimate topological index of reactivity (TIR) [16] indices describe in various extent the influence the nuclei motion, the molecular fragment conjugation, or the site with the maximum probability (entropy) in electrophilic substitution have on aromaticity viewed as increasing (for the first two indices) or decreasing (for the last index) as the more delocalized *π*-electrons in question, respectively. On the other hand, from the energetic perspective, the resonance energy (RE) or its version reported per concerned *π*-electrons (REPE) [22–24], together with the heat (enthalpy) of formation (as a thermodynamic stabilization criterion of energy) [25], give another alternative to quantify aromaticity rises with their increase (for the first two indices) and decrease (for the last index), respectively. Moreover, other groups of methods in aromaticity evaluation are developed by employing the molecular *magnetic* [17,26–30] as well as based on *electron delocalization* [31–37] properties. It is therefore clear that the aromaticity scale is neither unique in trend nor in quantification and deserves further geometrical-energetic comparative investigation.

In this context, wishing to provide a fresh geometrical vis-à-vis of energetic aromaticity discussion, the present paper introduces the atoms-in-molecules compactness form of aromaticity that is then specialized both at geometrical level though the polarizability information and within the energetic framework though the electronegativity and chemical hardness reactivity indices. Following this, they will be used for ordering ten basic organic compounds, aromatic annulens, amines, hydroxyarenes, and heterocycles with nitrogen, against the corresponding aromaticity within most common semi-empirical and *ab initio* quantum chemical methods. The present considerations and results aim to further clarify the relationship between the electronegativity and chemical hardness in modeling the molecular stability/reactivity/aromaticity, as well the “computational distance” among their output furnished by various quantum mechanical schemes used in structural chemistry. For all these, the aromaticity is involved both as the motif and the tool having overall the manifestly unifying character among the fundamental concepts and computational schemes of chemistry.

## 2. Methods

### 2.1. Quantum Compactness Aromaticity

Modeling the chemical bond is certainty key for describing the chemical reactivity and molecular structures’ stability. Yet, since the chemical bond is a dynamic state, for the best assessment of its connection with the stability and reactivity, the pre- and post-bonding stages are naturally considered.

**Figure 1.**
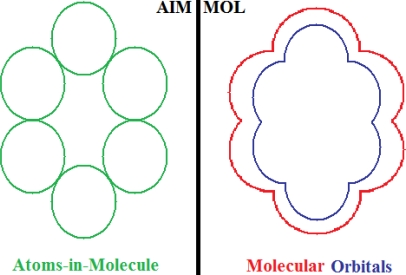
Heuristic representation of the concept of atoms-in-molecule (AIM) compactness aromaticity (for the benzene pattern) as the ratio of the pre-bonding atomic spheres’ based molecule to the (vis-à-vis) post-bonding molecular orbitals (MOL) modeling.

For the pre-bonding stage, the atomic spheres are considered in the atoms-in-molecule (AIM) arrangement, while for the post-bonding stage the molecular orbitals (MOL) of the already formed molecule are employed, see Figure 1; consequently, their ratio would model the *compactness degree* of a given property of AIM in respect to its counterpart at the MOL level of the chemical bond. Therefore, the actual compactness index of aromaticity and takes the general form

(1) $$\text{Aromaticity}=\frac{\Pi_{\text{AIM}}}{\Pi_{\text{MOL}}}\ldots \{\begin{array}{ll} \in (-\infty ,-1)\cup (1,+\infty ) & \ldots \text{AIM}\ldots \text{prevails}\\ \in (-1,+1) & \ldots \text{MOL}\ldots \text{prevails}\\ =\pm 1 & \ldots \text{transition}\ldots \text{states} \end{array}$$

that becomes workable once the property *Π* is further specified. Note that for the Equation (1) to be properly implemented, the chemical property *Π* should be equally defined and with the same meaning for the atoms and molecules, for consistency; such that what is compared is the chemical manifestation of the same property of bonding in its pre- or post-stage of formation. In other terms, Equation (1) may be regarded as a kind of “chemical limit” for the chemical bond that may be slightly oriented towards its atom constituents or to its molecular orbitals prescribing therefore the propensity to reactivity or stability, respectively.

It is worth considering also the quantitative difference between AIM and MOL properties of bonding, in which case the result may be regarded as the first kind of *absolute aromaticity*–for this reason, it is evaluated essentially between pre- and post-bonding stages and not relative to a referential (different) molecule [38], while the present approach promotes the *compactness* version of the aromaticity as the measure associated with the molecular stability in analog manner the compactness of rigid spheres in unit cells provides the crystal stability orderings. Yet, the proper scale hierarchy of compactness aromaticity, *i.e.*, the qualitative tendency respecting the quantitative yield of Equation (1), is to be established depending on the implemented chemical property. In what follows, both the geometrically- and energetically-based reactivity indices will be considered, and their associate AIM compactness aromaticity formula and scales formulated. Moreover, once various quantum methods in evaluating the MOL denominator property in Equation (1) are considered, they will become fully quantum.

### 2.2. Reactivity Indices-Based Aromaticity

#### 2.2.1. Geometric Index of Reactivity: Polarizability

Since it has been already shown [39] that the polarizability *α* of a conducting sphere of radius *r* is equal to *r*^3^, for the atomic dipole systems the induced perturbation on the electronic cloud the actual formula should be corrected as [40]:

(2) $$\alpha_{\text{Atom}}^{\text{Hati}-\text{Dutta}}=K\times r^{3}$$

with *K* a dipole related constant that has to be set out.

Historically, while a direct expansion of the Schrödinger differential equation in powers of *eigen-energy*, firstly done by Waller and Epstein [41,42], gives an analytic value of the polarizability of 4.5 a.u., the same value was found by variational method by Hassé through employing the Hydrogen ground state modified wave-function [43]

(3) $$\psi_{1s}^{*}=\psi_{1s}(1+Az+Bzr)$$

In modern times of quantum mechanics, the exact static dipole polarizabilities for the excited S states of the Hydrogen atom are determined by using the reduced free-particle Green's function method developed by McDowell and Porter [44] with the general formula for the polarizabilities found to be [45]:

(4) $$\alpha_{n}^{\text{McDowell}}=\frac{n^{4}({2n}^{2}+7)}{2}a_{0}^{3}$$

or with an even more general formula developed by Delone and Krainov [46] followed by Krylovetsky, Manakov, and Marmo [47]:

(5) $$\alpha_{\text{nl}}^{\text{DK}-\text{KMM}}=\frac{n^{4}}{{4Z}^{4}}[{4n}^{2}+7l(l+1)+14]a_{0}^{3}$$

The last two formulas give both the same celebrated Hydrogen static Polarizability of (9 / 2)*a*^3^_0_, where *a*_0_ is the Bohr radius.

Yet, another Hydrogenic Polarizability formulation can be actually elegantly developed from the first principles of quantum mechanics; it looks like (see Appendix for the complete derivation):

(6) $$\alpha_{\text{nl}}^{\text{Putz}}=\frac{a_{0}^{3}}{{2Z}^{2}}{\left[n^{2}(2+n^{2})-l^{2}{\left(1+l\right)}^{2}\right]}^{2}$$

that immediately recovers the Hydrogen exact limit:

(7) $$\alpha_{n=1,l=0}^{\text{Hydrogen}}=\frac{9}{2}a_{0}^{3}=0.667{\left[\overset{˚}{\text{A}}\right]}^{3}$$

Overall, from above discussion we can assume the universal atomic constant *K* = 4.5 for atoms, while the atoms-in-molecule polarizability may be written as the atoms in molecule superposition providing the contributing atomic radii is known:

(8) $$\alpha_{\text{AIM}}=\sum_{A}\alpha_{A}=4.5\sum_{A}r_{A}^{3}$$

The ansatz of summation of the atomic polarizabilities in molecular polarizability relays on the fact they associate with the deformation (or softness) property of electronic frontier distribution that is additive in overlapping phenomena.

On the other hand, for the molecular polarizability in post-bonding stage one may use the volume information:

(9) $$\alpha ∼r^{3}\Rightarrow \alpha ∼\frac{3}{4\pi }V{\left[\overset{˚}{\text{A}}\right]}^{3}$$

to advance the molecular (MOL) working expression throughout the normalizing factor involving the number of valence electrons:

(10) $$\alpha_{\text{MOL}}=\frac{3}{4\pi }\frac{1}{\left[\text{Valence}\;e^{-}\right]}V_{\text{MOL}}{\left[\overset{˚}{\text{A}}\right]}^{3}$$

that specializes for aromatics to the number of pi-electrons:

(11) $$\alpha_{\text{MOL}}^{\text{Aromatics}}=\frac{3}{4\pi }\frac{1}{\left[\pi e^{-}\right]}V_{\text{MOL}}{\left[\overset{˚}{\text{A}}\right]}^{3}$$

With atoms-in-molecule pre-bonding and the post-bonding molecular polarizabilities the related aromaticity geometrically based index may be constructed as their ratio:

(12) $$A_{\text{POL}}=\frac{\alpha_{\text{AIM}}}{\alpha_{\text{MOL}}}$$

The aromaticity scale is set upon the polarizability relation with the deformability power describing the molecular stability; as such, the higher MOL-polarizability respecting the AIM counterpart, the more flexible is the post-bonding molecular system against the external influences; consequently, *as A_POL_ decreases molecular stability increases*.

#### 2.2.2. Energetic Indices of Reactivity: Electronegativity and Chemical Hardness

In the same line as we proceeded with polarizability, we now set the AIM and MOL versions of energy based electronegativity and chemical hardness reactivity indices.

For electronegativity, the addition of atomic electronegativities *χ*_A_ in pre-bonding stage of a molecule is driven by the resumed formula [48,49]:

(13a) $$\chi_{\text{AIM}}=\frac{n_{\text{AIM}}}{\sum_{A}\frac{n_{A}}{\chi_{A}}}$$

where the total atoms in molecule *n_AIM_* is the sum of the *n_A_* atoms of each *A*-species present in the molecule:

(13b) $$\sum_{A}n_{A}=n_{\text{AIM}}$$

On the other hand, the electronegativity in the post-bonding stage of molecular state may be formulated through employing its relation with the total energy of a given system or state with *N* (valence) electrons [50–55], followed by the successive transformations:

(14) $$\begin{array}{c} \chi_{\text{MOL}}=-{\frac{\partial E}{\partial N}|}_{|N〉}\\ ≅\frac{E_{N+1}-E_{N-1}}{2}\\ =\frac{I_{1}+A_{1}}{2}\\ ≅-\frac{E_{\text{HOMO}(1)}+E_{\text{LUMO}(1)}}{2} \end{array}$$

by means of central difference approximation, the ionization potential and electronic affinity specializations:

(15a) $$I_{1}=E_{N-1}-E_{N}$$

(15b) $$A_{1}=E_{N}-E_{1+i}$$

while ending with considering the Koopmans’ frozen core approximation [56]

(16a) $$I_{1}≅-E_{\text{HOMO}(1)}$$

(16b) $$A_{1}≅-E_{\text{LUMO}(1)}$$

allowing therefore writing the MOL electronegativity in terms of highest occupied (HOMO) and lowest unoccupied (LUMO) frontier highest occupied and lowest unoccupied molecular orbitals, respectively.

The two forms of electronegativities, given by Equations (13) and (14), are next combined, according with the AIM-MOL aromaticity recipe of Equation (1), to provide the compactness electronegativity-based aromaticity index:

(17) $$A_{\text{EL}}=\frac{\chi_{\text{AIM}}}{\chi_{\text{MOL}}}$$

Now, the electronegativity reactivity principle under the form of electronegativity equalization in molecule [57,58] is used for establishing the behavior of the aromaticity scale based on Equation (17). Accordingly, though aromaticity is described as the ratio of AIM to MOL electronegativity, it is clear that the pre-bonding stage of atomic electronegativity equalization (electronic flowing) into the molecular unified orbitals is the dominant phenomena, so that it is expected to prevail. Therefore, the aromaticity index of electronegativity Equation (17) is higher as the molecular stability (formation) is better realized; in short, *as A_MOL_ increases, a more aromatic molecular system is assumed*.

For chemical hardness description, the AIM pre-bonding formulation happens to have the same analytical form as that found for the AIM electronegativity [59]:

(18) $$\eta_{\text{AIM}}=\frac{n_{\text{AIM}}}{\sum_{A}\frac{n_{A}}{\eta_{A}}}$$

while the MOL version is constructed based on the previous orbital energy prescriptions of Equations (15) and (16) as applied to the second order derivation of the total energy respecting to the total number of electrons in a given state towards the working HOMO-LUMO formulation [60–62]:

(19) $$\begin{array}{c} \eta_{MOL}=\frac{1}{2}{\frac{\partial^{2}E}{{\partial N}^{2}}|}_{|N〉}\\ ≅\frac{E_{N+1}-{2E}_{N}+E_{N-1}}{2}\\ =\frac{I_{1}-A_{1}}{2}\\ ≅\frac{E_{\text{LUMO}(1)}-E_{\text{HOMO}(1)}}{2} \end{array}$$

It nevertheless resembles the idea that as the frontier gap between the HOMO and the LUMO orbitals is larger as the molecular system is more stable, *i.e.*, less engaged into chemical reactions through its frontier electrons [63–66].

Combining Equations (18) and (19) into the general aromaticity definition of Equation (1) one has also the compactness chemical hardness-based aromaticity index

(20) $$A_{\text{Hard}}=\frac{\eta_{\text{AIM}}}{\eta_{\text{MOL}}}$$

with the scale trend fixed by the maximum hardness principle [59,67], abstracted from above MOL chemical hardness; in other words, the chemical reactivity described by chemical hardness is driven by the post-bonding stage in molecular formation requiring that a molecules is as stable as its HOMO-LUMO gap increases. Consequently, the aromaticity scale based on Equation (20) is arranged from the lowest to highest values that parallels the increasing reactivity and decreasing aromaticity; in short, *smaller A_Hard_, bigger aromaticity character for a molecular system*.

However, while atomic electronegativity and chemical hardness in evaluating AIM schemes of Equations (13) and (18) may be implemented by appealing various benchmarking scales [53,54,68], the molecular orbital energies in computing MOL counterparts Equations (14) and (19) require dedicated computations for each concerned molecule; as such, for better understanding and interpreting the obtained electronegativity- and chemical hardness-based aromaticity scales it is worth shortly reviewing the main quantum schemes mainly used in computing the (post-bonding) molecular spectra.

### 2.3. Quantum Methods for Molecular Orbitals

#### 2.3.1. General Mono-Electronic Orbitals’ Equations

Following the Dirac’s quote, once the Schrodinger equation:

(21) HΨ=EΨ

was established “The underlying physical laws necessary for the mathematical theory of a large part of physics and the whole of chemistry are thus completely known” [69].

Unfortunately, the molecular spectra based on the eigen-problem Equation (21) is neither directly nor completely solved without specific atoms-in-molecule and/or symmetry constraints and approximation. As such at the mono-electronic level of approximation the Schrodinger Equation (21) rewrites under the so called independent-electron problem:

(22) $$H_{i}^{\text{eff}}\psi_{i}=E_{i}\psi_{i}$$

with the aid of effective electron Hamiltonian partitioning:

(23) $$H=\sum_{i}H_{i}^{\text{eff}}$$

and the correspondent molecular monoelectronic wave-functions (orbitals) fulfilling the conservation rule of probability:

(24) $$\int \psi_{i}^{2}(\text{r})d\text{r}=1$$

However, when written as a linear combination over the atomic orbitals the resulted MO-LCAO wave-function:

(25) $$\psi_{i}=\sum_{\nu }C_{\nu i}\varphi_{\nu }$$

replaced in Equation (22) followed by integration over the electronic space allows for matrix version of Equation (22):

(26) $$\left(H^{\text{eff}})(C)=(S)(C)(E\right)$$

having the diagonal energy-matrix elements as the eigen-solution

(27a) $${\left(E\right)}_{ij}=E_{ij}=E_{i}\delta_{ij}=\{\begin{array}{ll} E_{i} & \ldots i=j\\ 0 & \ldots i\neq j \end{array}$$

to be found in terms of the expansion coefficients matrix (*C*), the matrix of the Hamiltonian elements:

(28) $$H_{\mu \nu }=\int \varphi_{\mu }H^{\text{eff}}\varphi_{\nu }d\tau$$

and the matrix of the (atomic) overlapping integrals:

(29) $$S_{\mu \nu }=\int \varphi_{\mu }\varphi_{\nu }d\tau$$

where all indices in Equations (27)–(29) refers to matrix elements since the additional reference to the “*i*” electron was skipped for avoiding the risk of confusion.

Yet, the solution of the matrix equation (26) may be unfolded through the Löwdin orthogonalization procedure [70,71], involving the diagonalization of the overlap matrix by means of a given unitary matrix (*U*), (*U*)^+^(*U*) =(1), by the resumed procedure:

(30a) $$\left(s)={\left(U\right)}^{+}(S)(U\right)$$

(30b) $${\left(s^{-1/2}\right)}_{ii}={\left[{\left(s\right)}_{ii}\right]}^{-1/2}$$

(30c) $$\left(S^{-1/2}\right)=\left(U\right)\left(s^{-1/2}\right){\left(U\right)}^{+}$$

(27b) $${\left(\left(S^{1/2}\right)\left(C\right)\right)}^{+}\left(\left(S^{-1/2}\right)\left(H^{\text{eff}}\right)\left(S^{-1/2}\right)\right)\left(\left(S^{1/2}\right)\left(C\right)\right)=\left(E\right)$$

However, the solution given by Equation (27b) is based on the form of effective independent-electron Hamiltonians that can be quite empirically constructed–as in Extended Hückel Theory [72]; such “arbitrariness” can be nevertheless avoided by the so called *self-consistent field* (SCF) in which the one-electron effective Hamiltonian is considered such that to depend by the solution of Equation (25) itself, *i.e.*, by the matrix of coefficients (*C*); the resulted “Hamiltonian” is called Fock operator, while the associated eigen-problem is consecrated as the Hartree-Fock equation:

(31) $${F\psi }_{i}=E_{i}\psi_{i}$$

In matrix representation Equation (31) looks like:

(32) (F((C)))(C)=(S)(C)(E)

that may be iteratively solved through diagonalization procedure starting from an input (*C*) matrix or–more physically appealing–from a starting electronic distribution quantified by the density matrix:

(33) $$P_{\mu \nu }=\sum_{i}^{occ}C_{\mu i}C_{i\nu }$$

with major influence on the Fock matrix elements:

(34) $$F_{\mu \nu }=H_{\mu \nu }+\sum_{\lambda \sigma }P_{\lambda \sigma }\left[\left(\mu \nu |\lambda \sigma )-\frac{1}{2}(\mu \lambda |\nu \sigma \right)\right]$$

Note that now the one-electron Hamiltonian effective matrix components *H_μV_* differ from those of Equation (28) in what they truly represent, this time the kinetic energy plus the interaction of a single electron with the core electrons around all the present nuclei. The other integrals appearing in Equation (34) are generally called the two-electrons-multi-centers integrals and are written as:

(35) $$\left(\mu \nu |\lambda \sigma \right)=\int \varphi_{\mu }^{A}({\text{r}}_{1})\varphi_{\nu }^{B}({\text{r}}_{1})\frac{1}{r_{12}}\varphi_{\lambda }^{C}({\text{r}}_{2})\varphi_{\sigma }^{D}({\text{r}}_{2}){d\text{r}}_{1}{d\text{r}}_{2}$$

From definition (35), there is immediate to recognize the special integral *J* = (*μμ*|*νν* as the Coulomb integral describing repulsion between two electrons with probabilities *φ_μ_*^2^ and *φ_ν_*^2^.

Moreover, the Hartree-Fock Equation (32) with implementations given by Equations (33)–(34) are known as Roothaan equations [73] and constitute the basics for closed-shell (or restricted Hartree-Fock, RHF) molecular orbitals calculations. Their extension to the spin effects provides the equations for the open-shell (or unrestricted Hartree-Fock, UHF) known also as the Pople-Nesbet Unrestricted equations [74].

#### 2.3.2. Semi-empirical Approximations

The second level of approximation in molecular orbital computations regards the various ways the Fock matrix elements of Equation (34) are considered, namely the approximations of the integrals (35) and of the effective one-electron Hamiltonian matrix elements *H_μV_*.

The main route for such an endeavor is undertaken through neglecting at different degrees certain *differential* overlapping terms (integrals)–as an offset ansatz–although with limited physical justification–while the adjustment with experiment is done (post-factum) by fitting parameters–from where the semi-empirical name of such approximation. Practically, by emphasizing the (nuclear) centers in the electronic overlapping integral (29):

(36) $$S_{\mu \nu }=\int \varphi_{\mu }^{A}({\text{r}}_{1})\varphi_{\nu }^{B}({\text{r}}_{1}){d\text{r}}_{1}$$

the differential overlap approximation may be considered by two situations.

- By *neglecting the differential overlap* (NDO) through the mono-atomic orbitalic constraint:
  (37a) $$\varphi_{\mu }\varphi_{\nu }=\varphi_{\mu }\varphi_{\mu }\delta_{\mu \nu }$$
  leaving with the simplified integrals:
  (37b) $$S_{\mu \nu }=\delta_{\mu \nu }\int \varphi_{\mu }^{A}({\text{r}}_{1})\varphi_{\mu }^{A}({\text{r}}_{1}){d\text{r}}_{1}=\delta_{\mu \nu }$$
  (37c) $$\left(\mu \nu |\lambda \sigma \right)=\delta_{\mu \nu }\delta_{\lambda \sigma }\int \varphi_{\mu }^{A}({\text{r}}_{1})\varphi_{\mu }^{A}({\text{r}}_{1})\frac{1}{r_{12}}\varphi_{\lambda }^{B}({\text{r}}_{2})\varphi_{\lambda }^{B}({\text{r}}_{2}){d\text{r}}_{1}{d\text{r}}_{2}=\delta_{\mu \nu }\delta_{\lambda \sigma }\left(\mu_{A}\mu_{A}|\lambda_{B}\lambda_{B}\right)\equiv \delta_{\mu \nu }\delta_{\lambda \sigma }\gamma^{AB}$$
  thus reducing the number of bielectronic integrals, while the tri- and tetra-centric integrals are all neglected;
- By *neglecting the diatomic differential overlap* (NDDO) of the bi-atomic orbitals:
  (38a) $$\varphi_{\mu }^{A}\varphi_{\nu }^{B}=\varphi_{\mu }^{A}\varphi_{\nu }^{A}\delta_{AB}$$
  that implies the actual simplifications:
  (38b) $$S_{\mu \nu }=\delta_{AB}\int \varphi_{\mu }^{A}({\text{r}}_{1})\varphi_{\nu }^{A}({\text{r}}_{1}){d\text{r}}_{1}=\delta_{AB}\delta_{\mu \nu }$$
  (38c) $$\left(\mu \nu |\lambda \sigma \right)=\delta_{AB}\delta_{CD}\int \varphi_{\mu }^{A}({\text{r}}_{1})\varphi_{\nu }^{A}({\text{r}}_{1})\frac{1}{r_{12}}\varphi_{\lambda }^{C}({\text{r}}_{2})\varphi_{\sigma }^{C}({\text{r}}_{2}){d\text{r}}_{1}{d\text{r}}_{2}$$
  when overlaps (or contractions) of atomic orbitals on different atoms are neglected.

For both groups of approximations specific methods are outlined below.

##### 2.3.2.1. NDO Methods

The basic NDO approximation was developed by Pople and is known as the Complete Neglect of Differential Overlap CNDO semi-empirical method [75–78]. It employs the approximation (37) such that the molecular rotational invariance is respected through the requirement the integral (37c) depends only on the atoms A or B where the involved orbitals reside–and not by the orbitals themselves. That is the integral γ*^AB^* in (37b) is seen as the average electrostatic repulsion between an electron in any orbital of A and an electron in any orbital of B:

(39) $$V_{AB}=Z_{B}\gamma^{AB}$$

In these conditions, the working Fock matrix elements of Equation (34) become within RHF scheme:

(40a) $$F_{\mu \mu }^{\text{CNDO}}=H_{\mu \mu }^{\text{CNDO}}+\left(P_{AA}-\frac{1}{2}P_{\mu \mu }\right)\gamma^{AA}+\sum_{B\neq A}P_{BB}\gamma^{AB}$$

(40b) $$F_{\mu \nu }^{\text{CNDO}}=H_{\mu \nu }^{\text{CNDO}}-\frac{1}{2}P_{\mu \nu }\gamma^{AB}$$

From Equations (40a&b) follows there appears that the core Hamiltonian has as well the diagonal and off-diagonal components; the diagonal one represents the energy of an electron in an atomic orbital of an atom (say A) written in terms of ionization potential and electron affinity of that atom [79]:

(41a) $${\text{U}}_{\mu \mu }^{\text{CNDO}}=-\frac{1}{2}\left(I_{\mu }+A_{\mu }\right)-\left(Z_{A}-\frac{1}{2}\right)\gamma^{AA}$$

added to the attraction energy respecting the other (B) atoms to produce the one-center-one-electron integrals:

(41b) $$H_{\mu \mu }^{\text{CNDO}}={\text{U}}_{\mu \mu }^{\text{CNDO}}-\sum_{B\neq A}V_{AB}$$

overall expressing the energy an electron in the atomic orbital *φ_μ_* would have if all other *valence electrons* were removed to infinity. The non-diagonal terms (*the resonance integrals*) are parameterized in respecting the overlap integral and accounts (through *β_AB_* parameter averaged over the atoms involved) on the diatomic bonding involved in overlapping:

(41c) $$H_{\mu \nu }^{\text{CNDO}}=\beta_{AB}^{\text{CNDO}}S_{\mu \nu }$$

The switch to the UHF may be eventually done through implementing the spin equivalence:

(41d) $$P^{T}\equiv P^{↑+↓}=\frac{1}{2}P^{↑}=\frac{1}{2}P^{↓}$$

although the spin effects are not at all considered since no exchange integral involved. This is in fact the weak point of the CNDO scheme and it is to be slightly improved by the next Semi-empirical methods.

The exchange effect due to the electronic spin accounted within the Intermediate Neglect of Differential Overlap (INDO) method [80] through considering in Equations (40a) and (41a) the exchange one-center integrals *γ^AA^* ≡ *K* = (*μν|μν*) is evaluated as:

(42) $${\left({sp}_{x}|{sp}_{x}\right)}^{\text{INDO}}=\frac{1}{3}G^{1},\;{\left(p_{x}p_{y}|p_{x}p_{y}\right)}^{\text{INDO}}=\frac{3}{25}F^{2}$$

in terms of the Slater-Condon parameters *G*^1^, *F*^2^, … usually used to describe atomic spectra.

The INDO method may be further modified in parameterization of the spin effects as developed by Dewar’s group and led with the Modified Intermediate Neglect of Differential Overlap (MINDO) method [75,81–89] whose basic equations look like:

(43a) $$F_{\mu \nu }^{↑(\text{MINDO})}=\{\begin{array}{ll} H_{\mu \nu }^{\text{MINDO}}-(\mu \mu |\nu \nu )P_{\mu \nu }^{↑} & \ldots {\mu |}_{A},\;{\nu |}_{B\neq A}\\ \left({2P}_{\mu \nu }^{↑+↓}-P_{\mu \nu }^{↑}\right)(\mu \nu |\mu \nu )-P_{\mu \nu }^{↑}(\mu \mu |\nu \nu ) & \ldots {\mu |}_{A}\neq {\nu |}_{A} \end{array}$$

(43b) $$F_{\mu \mu }^{↑(\text{MINDO})}=H_{\mu \mu }^{\text{MINDO}}+\sum_{\nu |A}\left[(\mu \mu |\nu \nu )P_{\nu \nu }^{↑+↓}-(\mu \nu |\mu \nu )P_{\nu \nu }^{↑}\right]+\sum_{B}\gamma_{\text{MINDO}}^{AB}\sum_{A}^{B}P_{\mu \mu }^{↑+↓}$$

Apart from specific counting of spin effects, another particularity of MINDO respecting the CNDO/INDO is that all the non-zero two-center Coulomb integrals are set equal and parameterized by the appropriate one center two electrons integrals *A_A_* and *A_B_* within the Ohno-Klopman expression [90,91]:

(44) $$\gamma_{\text{MINDO}}^{AB}=\left(s_{A}s_{A}|s_{B}s_{B}\right)=\left(s_{A}s_{A}|p_{B}p_{B}\right)=\left(p_{A}p_{A}|p_{B}p_{B}\right)=\frac{1}{\sqrt{r_{AB}^{2}+\frac{1}{4}{\left(\frac{1}{A_{A}}+\frac{1}{A_{B}}\right)}^{2}}}$$

The one-center-one-electron integral *H_μμ_* is preserved from the CNDO/INDO scheme of computation, while the resonance integral (41c) is modified as follows:

(45) $$H_{\mu \nu }^{\text{MINDO}}=\left(I_{\mu }+I_{\nu }\right)\beta_{AB}^{\text{MINDO}}S_{\mu \nu }$$

with the parameter $\beta_{AB}^{MINDO}$ being now dependent on the atoms-in-pair rather than the average of atomic pair involved. As in INDO, the exchange terms, *i.e.*, the one-center-two-electron integrals, are computed employing the atomic spectra and the *G^k^*, *F^k^*, Slater-Condon parameters, see Equation (42) [92]. Finally, it is worth mentioning that the MINDO (also with its MINDO/3 version) improves upon the CNDO and INDO the molecular geometries, heats of formation, being particularly suited for dealing with molecules containing heteroatoms.

##### 2.3.2.2. NDDO Methods

This second group of neglecting differential overlaps semi-empirical methods includes along the interaction quantified by the overlap of two orbitals centered on the same atom also the overlap of two orbitals belonging to different atoms. It is manly based on the Modified Neglect of Diatomic Overlap (MNDO) approximation of the Fock matrix, while introducing further types of integrals in the UHF framework [93–99]:

(46a) $$F_{\mu \nu }^{↑(\text{MNDO})}=\{\begin{array}{ll} H_{\mu \nu }^{\text{MNDO}}-\sum_{\lambda |A}\sum_{\sigma |B}(\mu \lambda |\nu \sigma )P_{\lambda \sigma }^{↑} & {\ldots \mu |}_{A},\;{\nu |}_{B\neq A}\\ H_{\mu \nu }^{\text{MNDO}}+P_{\mu \nu }^{↑}[(3(\mu \nu |\mu \nu )-(\mu \mu |\nu \nu )]+\sum_{B}\sum_{\lambda |B}\sum_{\sigma |B}(\mu \nu |\lambda \sigma )P_{\lambda \sigma }^{↑+↓} & {\ldots \mu |}_{A}\neq {\nu |}_{A} \end{array}$$

(46b) $$F_{\mu \mu }^{↑(\text{MNDO})}=H_{\mu \mu }^{\text{MNDO}}+\sum_{\nu |A}\left[(\mu \mu |\nu \nu )P_{\nu \nu }^{↑+↓}-(\mu \nu |\mu \nu )P_{\nu \nu }^{↑}\right]+\sum_{B}\sum_{\lambda |B}\sum_{\sigma |B}(\mu \mu |\lambda \sigma )P_{\lambda \sigma }^{↑+↓}$$

Note that similar expressions can be immediately written within RHF once simply replacing:

(47) $$P^{↑(↓)}=-\frac{1}{2}P^{↑+↓}$$

in above Fock (46a&b) expressions.

Now, regarding the (Coulombic) two-center-two-electron integrals of type (38c) appearing in Equations (46) there were indentified 22 unique forms for each pair of non-hydrogen atoms, *i.e.*, the rotational invariant 21 integrals (*ss*|*ss*), (*ss*|*p_σ_* *p_σ_*), (*ss*|*p_π_* *p_π_*),…,(*p_σ_* *p_σ_*|*p_σ_* *p_σ_*), (*p_π_* *p_π_*|*p_π_* *p_π_*),…, (*sp_σ_*|*sp_σ_*), (*sp_π_*|*sp_π_*),…, (*p_π_* *p_σ_*|*sp_π_*), (*p_π_* *p_σ_*|*p_π_* *p_σ_*), and the 22^nd^ one that is written as a combination of two of previously ones, namely (*p_π_* *p_π_*′|*p_π_* *p_π_*′) = 0.5[(*p_π_* *p_π_*|*p_π_* *p_π_*) – (*p_π_* *p_π_|p_π_*′ *p_π_*′)], with the typical integral approximation relaying on the Equation (44) structure, however slightly modified as:

(48) $${\left(ss|ss\right)}^{\text{MNDO}}=\frac{1}{\sqrt{{\left(r_{AB}+c_{A}+c_{B}\right)}^{2}+\frac{1}{4}{\left(\frac{1}{A_{A}}+\frac{1}{A_{B}}\right)}^{2}}}$$

where additional parameters *c_A_* and *c_B_* represent the distances of the multipole charges from their respective nuclei. The MNDO one-center one-electron integral has the same form as in NDO methods, *i.e.*, given by Equation (41b) with the average potential of Equation (39) acting on concerned center; still, the resonance integral is modified as:

(49) $$H_{\mu \nu }^{\text{MNDO}}=\frac{\beta_{\mu }^{\text{MNDO}}+\beta_{\nu }^{\text{MNDO}}}{2}S_{\mu \nu }$$

containing the atomic adjustable parameters $\beta_{\mu }^{MNDO}$ and $\beta_{\nu }^{MNDO}$ for the orbitals *φ_μ_* and *φ_ν_* of the atoms A and B, respectively. The exchange (one-center-two-electron) integrals are mostly obtained from data on isolated atoms [79]. Basically, MNDO improves MINDO through the additional integrals considered the molecular properties such as the heats of formations, geometries, dipole moments, HOMO and LUMO energies, *etc*., while problems still remaining with four-member rings (too stable), hypervalent compounds (too unstable) in general, and predicting out-of-plane nitro group in nitrobenzene and too short bond length (∼ 0.17 Å) in peroxide–for specific molecules.

The MNDO approximation is further improved by aid of the Austin Model 1 (AM1) method [100–102] that refines the inter-electronic repulsion integrals:

(50) $${\left(s_{A}s_{A}|s_{B}s_{B}\right)}^{AM1}=\frac{1}{\sqrt{r_{AB}^{2}+\frac{1}{4}{\left(\frac{1}{{AM}_{A}}+\frac{1}{{AM}_{B}}\right)}^{2}}}$$

while correcting the one-center-two-electron atomic integrals of Equation (44) by the specific (AM) monopole-monopole interaction parameters. In the same line, the nuclei-electronic charges interaction adds an energetic correction within the *α_AB_* parameterized form:

(51) $${\Delta E}_{AB}=\sum_{A,B}\left\{Z_{A}Z_{B}\left(s_{A}s_{A}|s_{B}s_{B}\right)\left[1+\left(1+\frac{1}{r_{AB}}\right)e^{-\alpha_{AB}r_{AB}}\right]-Z_{A}Q_{B}\left(s_{A}s_{A}|s_{B}s_{B}\right)\right\}$$

The AM1 scheme, while furnishing better results than MNDO for some classes of molecules (e.g., for phosphorous compounds), still provides inaccurate modeling of phosphorous-oxygen bonds, too positive energy of nitro compounds, while the peroxide bond is still too short. In many case the reparameterization of AM1 under the Stewart’s PM3 model [103,104] is helpful since it is based on a wider plethora of experimental data fitting with molecular properties. The best use of PM3 method lays in the organic chemistry applications.

To systematically implement the transition metal orbitals in semi-empirical methods the INDO method is augmented by Zerner’s group either with non-spectroscopic and spectroscopic (*i.e.,* fitting with UV spectra) parameterization [105–107], known as ZINDO/1 and ZINDO/S methods, respectively [108–114]. The working equations are formally the same as those for INDO except for the energy of an atomic electron of Equation (41a) that now uses only the ionization potential instead of electronegativity of the concerned electron. Moreover, for ZINDO/S the core Hamiltonian elements *H_μμ_* is corrected:

(52) $${\Delta H}_{\mu \mu }^{\text{ZINDO}}=\sum_{B}\left(Z_{B}-Q_{B}\right)\gamma_{\left(\mu \mu |ss\right)}^{AB(\text{ZINDO})}$$

by the *f_r_* parameterized integrals:

(53) $$\gamma_{\left(\mu \mu |ss\right)}^{AB(\text{ZINDO})}=\frac{f_{r}}{\frac{{2f}_{r}}{\gamma_{\mu \mu }^{A}+\gamma_{ss}^{B}}+r_{AB}},\;\;f_{r}=1.2$$

in terms of the one-center-two-electron Coulomb integrals $\gamma_{\mu \mu }^{A}$, $\gamma_{ss}^{B}$. Equation (53) conserves nevertheless the molecular rotational invariance through making the difference between the *s*- and *d*-Slater orbitals exponents. The same types of integrals correct also the nuclei-electronic interaction energy by quantity:

(54) $${\Delta E}_{AB}=\sum_{A,B}\left\{\frac{Z_{A}Z_{B}}{r_{AB}}-Z_{A}Q_{B}\gamma_{\left(\mu \mu |ss\right)}^{AB}\right\}$$

Since based on fitting with spectroscopic transitions the ZINDO methods are recommended in conjunction with single point calculation and not with geometry optimization, this should be consider by other off-set algorithms.

Beyond either NDO or NDDO methods, the self-consistent computation of molecular orbitals can be made by the so called *ab initio* approach, directly relaying on the HF equation or on its density functional extension, as will be in next sketched.

#### 2.3.3. *Ab initio* Methods

The alternative to semi-empirical methods is the full self-consistent calculation or the so called *ab initio* approach; it is based on computing of all integrals appearing on Equation (34), yet with the atomic Slater type orbitals (STO), exp(−αr), being replaced by the Gaussian type orbitals (GTO) [115]:

(55a) $$\varphi_{A}^{\text{GTO}}=x_{A}^{l}y_{A}^{m}z_{A}^{n}\text{exp}\left(-{\alpha r}_{A}^{2}\right)$$

in molecular orbitals expansion–a procedure allowing for much simplification in multi-center integrals computation. Nevertheless, at their turn, each GTO may be generalized to a contracted expression constructed upon the primitive expressions of Equation (55a):

(55b) $$\varphi_{\mu }^{\text{CGTO}}(r_{A})=\sum_{p}d_{p\mu }\varphi_{p}^{\text{GTO}}\left(\alpha_{p},\;r_{A}\right)$$

where *d_pμ_* and *α_A_* are called the exponents and the contraction coefficients of the primitives, respectively. Note that the primitive Gaussians involved may be chosen as approximate Slater functions [116], Hartree-Fock atomic orbitals [117], or any other set of functions desired so that the computations become faster. In these conditions, a minimal basis set may be constructed with one function for H and He, five functions for Li to Ne, nine functions for Na to Ar, 13 functions for K and Ca, 18 functions for Sc to Kr,..., *etc*., to describe the core and valence occupancies of atoms [118–120]. Although such basis does not generally provide accurate results (because of its small cardinal), it contains the essential information regarding the chemical bond and may be useful for qualitative studies, as is the present case for aromaticity scales where the comparative trend is studied.

##### 2.3.3.1. Hartree-Fock Method

When simple *ab initio* method is referred it means that the Hartree-Fock equation (31) with full Fock matrix elements [121–124] of Equations (33) and (34) is solved for a Gaussian contracted basis (55). Actually, the method evaluates iteratively the kinetic energy and nuclear-electron attraction energy integrals–for the effective Hamiltonian, along the overlap and electron-electron repulsion energy integrals (for both the Coulomb and exchange terms), respectively written as:

(56a) $$T_{\mu \nu }=〈\mu |\left(-\frac{1}{2}\nabla^{2}\right)|\nu 〉$$

(56b) $$V_{\mu \nu }=〈\mu |\frac{Z_{A}}{r_{A}}|\nu 〉$$

(56c) $$S_{\mu \nu }=〈\mu |\nu 〉$$

(56d) $$\left(\mu \nu |\lambda \sigma )=(\mu \nu |\frac{1}{r_{12}}|\lambda \sigma \right)$$

until the consistency in electronic population of Equation (33) between two consecutive steps is achieved.

Note that such calculation assumes the total wave function as a single Slater determinant, while the resultant molecular orbital is described as a linear combination of the atomic orbital basis functions (MO-LCAO). Multiple Slater determinants in MO description projects the configurationally and post-HF methods, and will not be discussed here.

##### 2.3.3.2. Density Functional Theory Methods

The main weakness of the Hartree-Fock method, namely the lack in correlation energy, is ingeniously restored by the Density Functional method through introducing of the so called effective one-electron exchange-correlation potential, yet with the price of not knowing its analytical form. However, the working equations have the simplicity of the HF ones, while replacing the exchange term in Equation (34) by the exchange-correlation (“XC”) contribution; there resulted the (general) unrestricted matrix form of the Kohn-Sham equations [125]:

(57a) $$F_{\mu \nu }^{↑}=H_{\mu \nu }^{↑}+\sum_{\lambda \sigma }P_{\lambda \sigma }^{T}(\mu \nu |\lambda \sigma )+F_{\mu \nu }^{XC↑}$$

(57b) $$F_{\mu \nu }^{↓}=H_{\mu \nu }^{↓}+\sum_{\lambda \sigma }P_{\lambda \sigma }^{T}(\mu \nu |\lambda \sigma )+F_{\mu \nu }^{XC↓}$$

(58) $$P^{T}\equiv P^{↑+↓}=P^{↑}+P^{↓}$$

in a similar fashion with the Pople-Nesbet equations of Hartree-Fock theory. The restricted (closed-shell) variant is resembled by the density constraint:

(59) $$\rho^{↑}=\rho^{↓}$$

in which case the Roothaan analogous equations (for exchange-correlation potential) are obtained.

Either Equations (57a) or (57b) fulfils the general matrix equation of type (32) for the energy solution:

(60) $$E=\sum_{\mu \nu }P_{\mu \nu }H_{\mu \nu }+\frac{1}{2}\sum_{\mu \nu \lambda \sigma }P_{\mu \nu }P_{\lambda \sigma }(\mu \nu |\lambda \sigma )+E_{XC}$$

that can be actually regarded as the solution of the Kohn-Sham equations themselves. The appeared exchange-correlation energy *E_XC_* may be at its turn conveniently expressed through the energy density (per unit volume) by the integral formulation:

(61) $$E_{XC}=E_{XC}\;[\rho^{↑},\;\rho^{↓}]=\int f(\rho^{↑},\;\rho^{↓})d\tau$$

once the Fock elements of exchange-correlation are recognized to be of density gradient form [126]:

(62) $$F_{\mu \nu }^{XC↑(↓)}=\int \frac{\partial f}{\partial \rho^{↑(↓)}}\varphi_{\mu }\varphi_{\nu }d\tau$$

The quest for various approximations for the exchange-correlation energy density *f*(*ρ*) had spanned the last decades in quantum chemistry, and was recently reviewed [66]. Here we will thus present the “red line” of its implementation as will be further used for the current aromaticity applications. The benchmark density functional stands the Slater exchange approximation, derived within the so called Xα theory [127]:

(63a) $$f^{X\alpha }=-\frac{9}{4}\alpha {\left(\frac{3}{4\pi }\right)}^{1/3}\left(\rho^{↑4/3}+\rho^{↓4/3}\right)$$

with the *α* taking the values:

(63b) $$\alpha =\{\begin{array}{ll} 1 & \ldots \text{Slater}\\ 2/3 & \ldots \text{uniform}\;\text{electron}\;\text{gas} \end{array}$$

With Equation (63) in Equation (62) the resulted Kohn-Sham “exchange-correlation” matrix elements (although rooting only in the exchange) yields the integral representation:

(64) $$F_{\mu \nu }^{XC↑}=F_{\mu \nu }^{X↑}=-\frac{9}{4\pi }\alpha \int \rho^{↑1/3}\varphi_{\mu }\varphi_{\nu }d\tau$$

Considerably improvement for molecular calculation was given by Becke’s density gradient correction of the local spin density (or Slater exchange) approximation of the exchange energy [128]:

(65) $$E_{X}=\sum_{\sigma =↑,↓}\int e_{X\sigma }^{\text{LSDA}}g_{X\sigma }d\tau$$

where

(66) $$g_{X\sigma }\left(\frac{\left|{\nabla \rho }^{\sigma }\right|}{\rho^{\sigma 4/3}}\right)=g_{X\sigma }(x)=\{\begin{array}{ll} 1 & \ldots \text{Slater}\\ 1+\frac{{bx}^{2}}{a(1+6bx{\text{sinh}}^{-1}x)} & \ldots \text{Becke}88 \end{array}$$

with the parameters *a* = (3/2)(3/4*π*)^1/3^ and *b* = 0.0042 chosen to fit the experiment. Other exchange functionals were developed along the same line, *i.e.*, having different realization of the gradient function (66), most notable being those of Perdew and collaborators (e.g., Perdew-Wang-91, PW91) [129].

The correlation contribution was developed on a somewhat different algorithm, namely employing its definition as the difference between the exact and Hartree-Fock (HF) total energy of a polyelectronic system [130]. Without reproducing the results (more detailed are provided in the dedicated review of Ref. [66]), for the actual purpose we mention only the Lee-Yang-Parr (LYP) correlation functional [131–133] along the Vosko-Wilk-Nusair (VWN) local correlation density functional [134].

However, the exchange and correlation density functionals combine into the so called hybrid functionals; those used in the present study refer to:

- B3-LYP: advanced by Becke by empirical comparisons against very accurate results and contains the exchange contribution (20% HF + 8% Slater + 81% Becke88) added to the correlation energy (81% LYP + 19% VWN) [135];
- B3-PW91: was developed also by Becke with PW91 correlation instead of LYP;
- EDF1: was optimized for a specific basis set (6–31 + G^*^) and represent a rearrangement of Becke88 with LYP functionals with slightly different parameters, being an improvement over B3-LYP and Becke88-LYP combinations;
- Becke-97: is a hybrid exchange-correlation functional appeared by extending the *g*(*x*) of Equation (66) as a power series containing three terms with an admixture of 19.43% HF exchange [136].

These are the main methods, at both conceptual and computational levels, to be in next used to asses and compare the atoms-in-molecule compactness aromaticity scales for basics organics.

## 3. Application on Basic Aromatics Scales

The above reactivity indices-based aromaticity scales are now computed within the presented quantum chemical schemes for a limited yet significant series of benzenoids (see Table 2) containing the “life” atoms of Table 1. The atoms-in-molecule of aromaticity scales of polarizability, electronegativity and chemical hardness of Equations (12), (17), and (20) are directly computed upon the formulas given by Equations (8), (13), and (18), respectively; they are based exclusively on the data of Table 1 with the AIM results listed in Table 2, the 5th, 9th, and 10th columns, respectively.

For the post-bonding evaluations of the same indices, one must note the special case of polarizability that is computed upon the general Equation (11)–thus involving the molecular volume pre-computation. Here it is worth commenting on the fact that one can directly compute the molecular polarizability in various quantum schemes–however, with the deficiency that such procedure does not distinguishes among the stereo-isomers, *i.e.*, molecules VII (1-Naphthol) and VIII (2-Naphthalelon); IX (2-Naphthalenamine) and X (1-Naphthalenamine) in Table 2, since furnishing the same values, respectively; instead the same quantum scheme is able to distinguish between the volumes of two stereo-isomers making the Equation (11) as a more general approach. This way, the molecular volumes are reported in the 6th column of Table 2 as computed within the *ab initio*–Hartree Fock method of Section 2.3.3.1; note that the HF method was chosen as the reference since it is at the “middle computational distance” between the semi-empirical and density functional methods; it has only the correlation correction missing; however, even the density functional schemes, although encompassing in principle correlation along the exchange–introduces approximations on the last quantum effect. Therefore, the molecular polarizability is computed upon the Equation (11) in the 7th column of Table 2 with the associate polarizability compactness aromaticities displayed in the 8th column of Table 2.

**Figure 2.**
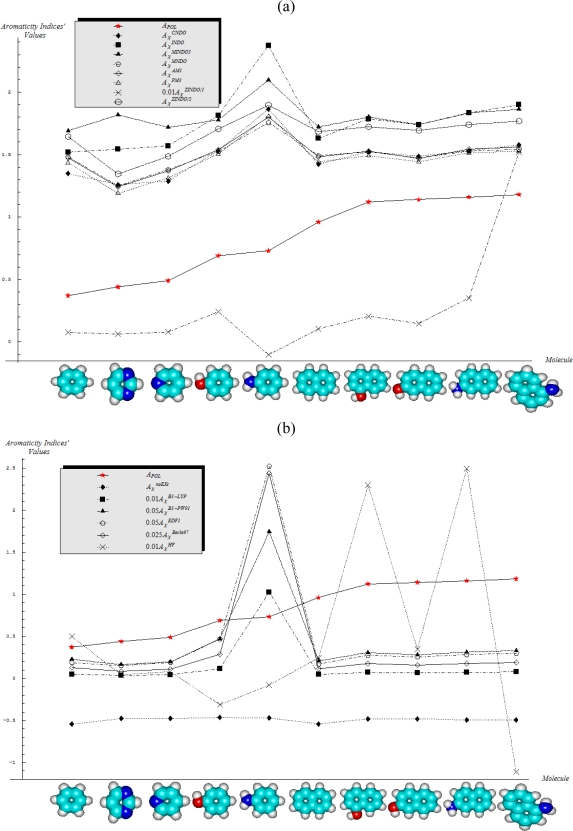
Electronegativity-based aromaticity scales of Tables 3 and 4 computed within semi-classical schemes in (**a**) and within *ab initio* schemes in (**b**), as compared with the polarizability-based aromaticity scale of Table 2, respectively.

**Figure 3.**
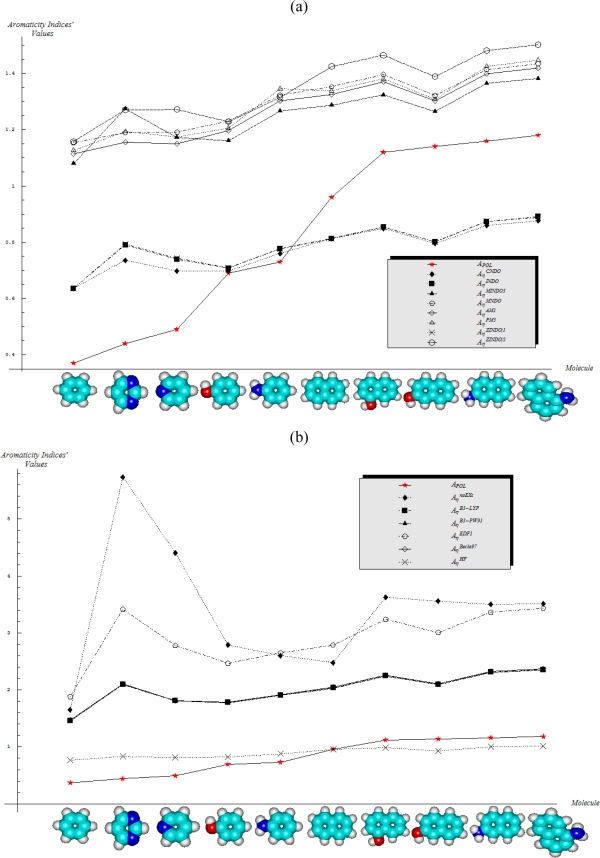
The chemical hardness-based aromaticity scales of Tables 3 and 4 computed within semi-classical schemes in (**a**) and within *ab initio* schemes in (**b)**, respectively.

The molecular energetic reactivity indices of electronegativity and chemical hardness are computed upon the Equations (14) and (19) in terms of HOMO and LUMO energies computed within the quantum semi-empirical and *ab initio* methods presented in Section 2.3; their individual values as well as the resulted quantum compactness aromaticities, when combined with the AIM values of Table 2, in Equations (17) and (20) are systematically communicated in Tables 3 and 4, with adequate scaled representations in Figures 2 and 3, respectively.

Note that neither the minimal basis set (STO-3G) nor the single point computation frameworks, although both motivated in the present context in which only the bonding and the post-bonding information should be capped in computation, does not affect the foregoing discussion by two main reasons: (i) they have been equally applied for all molecules considered in all quantum methods’ combinations; and (ii) what is envisaged here is the aromaticity trend, *i.e.*, the intra- and inter- scales comparisons rather than the most accurate values since no exact or experimental counterpart available for aromaticity.

Now, because of the observational quantum character of polarizability, one naturally assumes the (geometric) polarizability based- aromaticity scale of Table 2 as that furnishing the actual standard ordering among the considered molecules in accordance with the rule associated with Equation (12); it features the following newly introduced rules along possible generalizations

***Aroma1 Rule***: *the mono-benzenoid compounds have systematically higher aromaticity than those of double-ring benzenoids*; yet, this is the generalized version of the rule demanding that the benzene aromaticity is always higher than that of naphthalene, for instance; however, further generalization respecting the poly-ring benzenoids is anticipated albeit it should be systematically proved by appropriate computations;
***Aroma2 Rule***: *C-replaced benzenoids are more aromatic than substituted benzenoids*, e.g., Pyridine and Pyrimidine vs. Phenol and Aniline ordering aromaticity in Table 2; this rule extends the substituted *versus* addition rules in aromaticity historical definition (see Introduction);
***Aroma3 Rule***: *double-C-replaced annulens have greater aromaticity than mono-C-replaced annulenes*, e.g., A_Pyrimidine_ > A_Pyridine_; this is a sort of continuation of the previous rule in the sense that as more Carbons are replaced in aromatic rings, higher aromaticity is provided; further generalization to poly-replacements to poly-ring benzenoids is also envisaged;
***Aroma4 Rule***: *hydroxyl-substitution to annulene produces more aromatic (stable) compounds than the correspondent amine-substitution*; e.g., this rule is fulfilled by mono-benzenoids and is maintained also by the double-benzene-rings no matter the stereoisomers considered; due to the fact the *π* electrons provided by Oxygen in hydroxyl-group substituted to annulene ring is greater than those released by Nitrogen in annulene ring by the amine-group substitution this rule is formally justified, while the generalization for hydroxyl- versus amine- substitution to poly-ring annulens may be equally advanced for further computational confirmation;
***Aroma5 Rule***: *for double ring annulens the* *α* *position is more aromatic for hydroxyl-substitution while* *β* *position is more aromatic for amine-substitution than their* *β* *and* *α* *counterparts*, *respectively*; this rule may be justified in the light of the Aroma4 Rule above employing the inverse role the Oxygen and Nitrogen plays in furnished (*π* + free pair) electrons to annulens rings: while for Oxygen the higher atomic charge may be positioned closer to the common bond between annulens’ rings–thus favoring the alpha position, the lesser Nitrogen atomic charge should be located as much belonging to one annulene ring only–thus favoring the beta position; such inversion behavior is justified by the existing of free electrons on the NH_2_- group that as closely are to the benzenic ring as much favors its stability against further electrophilic attack–as is the case of beta position of 2-Naphtalenamine in Table 2; extensions to the poly-ring annulens may be also investigated.

Under the reserve that these rules and their generalizations should be verified by extra studies upon a larger set of benzenoid aromatics, we will adopt them here in order to analyze their fulfillment with the energetically-based aromaticity scales of electronegativity and chemical hardness, reported in Tables 3 and 4 and drawn in Figures 2 and 3; actually, their behavior is analyzed against the aromaticity ordering rules given by Equations (17) and (20), i.e., as being anti-parallel and parallel with the polarizability-based aromaticity trend of Equation (12), with the results systematized in Tables 5 and 6, respectively.

From Table 5 there follows that electronegativity based-aromaticity displays the following properties respecting the aromaticity rules derived from polarizability framework:

- No semi-empirical quantum method, in general, satisfies the first rule of aromaticity, Aroma1, in the sense that the trend in Figure 2a (and Table 3) displays rather growing aromaticity character from mono- to double-benzenoid rings; the same behavior is common also to HF computational environment, perhaps due to the close relationships with approximations made in semi-empirical approaches; instead, all other *ab initio* methods considered, including that without exchange and correlation terms in Equations (57), do fulfill the Aroma1 rule;
- The remaining aromaticity Aroma2–5 rules are generally not adapted with any of the semi-empirical methods, except the MINDO3 (the most advanced and accurate method from the NDO approximations) fulfilling the Aroma3 rule regarding the ordering of mono- *versus* bi- CH- replacement group by Nitrogen on benzenoid ring. Interestingly, the Aroma3 rule is then not satisfied by any of the *ab initio* quantum methods;
- Aroma2 rule about the comparison between the CH- replacement group and the H- substitution to the mono ring benzene seems being in accordance only with HF and *ab initio* without exchange-correlation environments leading with the idea the electronegativity based- aromaticity of substitution and replacement groups is not so sensitive to the spin and correlation effects, being of primarily Coulombic nature;
- Hydroxyl- *versus* amine- substitution aromaticity appears that is not influenced by spin and correlation in electronegativity based- ordering aromaticity since only the no-exchange and correlation computational algorithm agrees with Aroma4 Rule;
- α- *versus* β- stereoisomeric position influence in aromaticity ordering is respected only by the HF scheme of computation and by no other combination, either semi-empirical or *ab initio*.

Overall, it seems electronegativity may be used in modeling compactness of atoms-in-molecules aromaticity–basically without counting on the exchange or correlation effects, or at best within the HF algorithm, while semi-empirical methods seems not well adequate. Yet, for all aromaticity rules formulated, there exists at least one quantum computational environment for which the electronegativity based compactness aromaticity is in agree with each of them.

The situation changes significantly when chemical hardness is considered for compactness aromaticity computation; the specific behavior is abstracted from the analysis of Table 6 and can be summarized as follows:

- Semi-empirical methods are equally appropriate in producing agreement with Aroma1 and Aroma4 rules in what concerns the aromaticity behavior for the mono- *versus* bi- ring annulens and hydroxyl- *versus* amine- substitution to either of them, respectively;
- Aroma2 and Aroma3 rules are slightly better fulfilled by the semi-empirical than the *ab initio* quantum frameworks in modeling the aromaticity performance of the mono- *versus* bi- CH- replaced groups and both of them against the H- substituted on benzenic rings, respectively;
- The stereoisomeric effects comprised by the Aroma5 rule is not modeled by the chemical hardness compactness aromaticity by any of its computed scales, neither semi-empirical or *ab initio*.

Overall, when the chemical hardness agrees with one of the above enounced Aroma Rules it does that within more than one computational scheme; however, the best agreement of chemical hardness with polarizability based- aromaticity scales is for the mono- *versus* bi- (and possible poly-) benzenic rings decreasing of aromaticity orderings, along the manifestly hydroxyl- superior effects in aromaticity than amine- groups substitution within most of the computational quantum schemes, *i.e.*, valid either for semi-empirical and *ab initio* methods. The stereoisomerism is not covered by chemical hardness modeling aromaticity, and along the electronegativity limited coverage within HF scheme in Table 5, there follows that the energetic reactive indices are not able to prevail over the geometric indices as polarizability or to predict stereoisomerism ordering in aromaticity modeling compactness schemes.

Finally, few words about the output of the various quantum computational schemes respecting the current aromaticity definition given by Equation (1) are worth addressing. As such, one finds that:

- With CNDO and INDO methods, the electronegativity based-aromaticity is more oriented towards the AIM limit of Figure 1, while chemical hardness based- aromaticity merely models the MOL limit of chemical bonding, see Table 3. This agrees with the basic principles of chemical reactivity according to which electronegativity drives the atomic encountering in forming the transition state towards chemical bond, while chemical hardness refines the bond by the aid of maximum hardness principle [59,67];
- The MINDO3, MNDO, AM1, PM3, and ZINDO/S all display in Table 3 the exclusively AIM limit in assessing aromaticity in bonding, yet with electronegativity based values systematically higher than those based on chemical hardness–this way respecting in some degree the empirical rule stating that the electronegativity stands as the first order effect in reactivity, while the chemical hardness corrects in the second order the bonding stability, according with the basic differential definitions of Equations (14) and (19), respectively;
- ZINDO/1 differs both from ZINDO/S and by the rest of semi-empirical methods of the last group, while giving qualitative results in the same manner as CNDO and INDO, in the sense of higher absolute (positively defined) electronegativity based-respecting the chemical hardness based-aromaticities, yet with significant quantitative values over unity (*i.e.*, the transition state as the instable equilibrium between AIM and MOL limits), see Table 3. This means that the transitional elements’ orbitals inclusion without further refinements of ZINDO/S exacerbates the Coulombic atoms-in-molecule effects, *i.e.*, the stability (aromaticity) of bonding is mostly to be acquired in the pre-bonding stage of the AIM limit;
- Somehow with the same qualitative-quantitative behavior as ZINDO/1 is the HF computed aromaticities indices of Table 4; however, the negative values as well as exceeding the AIM unity limit of electronegativity based-aromaticities appear now as multiple-recordings, while the resulted chemical hardness aromaticity is the closest respecting the unity limit of transition state prescribed by Equation (1). Together, this information shows that the HF computational framework merely models the pre-bonding AIM and the post-bonding MOL stages by electronegativity and chemical hardness reactivity indices, respectively;
- The reverse case to HF computing stands the no-exchange-and-correlation (noEX-C) values in Table 4, according to which the electronegativity based aromaticity, beside the negative values, are all in sub-unity range, so being associated with post-bonding MOL limit. This corroborates the situation with the supra-unitary recordings of chemical hardness based-aromaticity outputs, specific to pre-bonding AIM, the resulted reactivity picture is completely reversed respecting that accustomed for electronegativity and chemical hardness reactivity principles [54]. Therefore, it is compulsory to consider at least the electronic spin through exchange contributions (as in the HF case), not only conceptually, but also computationally for achieving a consistent picture of reactivity, not only of the aromaticity;
- The last situation is restored by using the hybrid functionals of DFT, *i.e.*, B3-LYP, B3-PW91, EDF1, and Becke97 in Table 4, with the help of which electronegativity based-aromaticity regains its supremacy over that computed with the chemical hardness AIM and MOL limits in bonding. Although, no explicit sub-unity MOL limit of Equation (1) is obtained with chemical hardness aromaticity computation, the recorded values are enough close to unity, while those based on electronegativity are more than twice further away from unity, to can say that the reactivity principles are fairly respected within these quantum methods, *i.e.*, when *A_χ_* and *A_η_* are situated in the AIM and MOL limiting sides of chemical bonding, respectively.

The bottom line is rising by the wish to globally combine the ideas of quantum chemical methods used in chemical bonding, reactivity principles, and aromaticity results; upon the above discussions it follows that MINDO3, AM1 (or PM3)–for semi-empirical along Becke hybrid functionals and Hartree-Fock–for *ab initio* are the suited methods that fulfils most of the reactivity and the present introduced aromaticity bonding rules. However, the best of them overall seems to remain the consecrated HF scheme, since acquiring the highest number of grades summated throughout Tables 5 and 6. As such, a new challenge appears since the present results recommend that correlation does not count too much in aromaticity or reactivity modeling. Nevertheless, further studies with larger set of molecules and types of aromatics should be address for testing whether or not the advanced aromaticity (Aroma1–5) rules are preserved or in which degree they may be generalized or modified such that being in accordance with the principles of chemical bonding and reactivity.

## 4. Conclusions

Modeling the stability and reactivity of molecules is perhaps the greatest challenge in theoretical and computational chemistry. This is because the main conceptual tools developed as the reactivity indices of electronegativity and chemical hardness along the associate principles are often suspected by the lack of observability character. Therefore, although very useful in formal explanations of chemical bonding and reactivity, it is hard to find their experimental counterpart unless expressed by related measurable quantities as energy, polarizability, refractivity, *etc*. When the aromaticity concept come into play, it seems no further conceptual clarification is acquired, since no quantum observable or further precise definition can be advanced; in fact, the aromaticity concept associates either with geometrical, energetic, topologic, electronic molecular circuits (currents), or with the less favored entropic site in a molecule, just to name few of its representations.

However, since at the end, the aromaticity appears to describe the stability character of the molecular sample, its connection with a reactivity index seems natural, although systematically ignored so far. In this respect, the present work focuses on how the electronegativity and chemical hardness based-aromaticity scales behave with respect to others constructed on a direct observable quantum quantity–the polarizability in this case. This is because the polarizability quantity is fundamental in quantum mechanics and usually associated with the second order Stark effect that can be computed within the perturbation theory (see Appendix). Then, two ways of seeing a molecular structure were employed in introducing the actual absolute aromaticity definition:

- *the molecule viewed as composed of the constituent atoms* (*AIM*) and
- *the molecule viewed from its spectra of molecular orbitals* (*MOL*).

The two molecular perspectives may be associated with the pre- and post-bonding stages of a chemical bond at equilibrium; therefore, the conceptual and computational competition between these two molecular facets should measure the stability or its contrary effect - the reactivity propensity - being therefore the ideal ingredients for an absolute definition of aromaticity. Note that although an AIM-to-MOL *difference definition* of absolute aromaticity was recently advanced [38], the actual study of their *ratio definition* should account for a sort of compactness degree of molecular structure–as described by the specific molecular property used.

In short, for a molecular property to become a candidate for absolute (here with its compactness variant of) aromaticity, it has to fulfill two basic conditions:

- *having a viable quantum definition* (since the quantum nature of electrons and nucleus are assumed as responsible for molecular stability/reactivity/aromaticity); and
- *having a reality at both the atomic and molecular levels*.

In this respect, all the presently considered reactivity indices, *i.e.*, polarizability, electronegativity, and chemical hardness, have equally consecrated quantum definitions as well as atomic and molecular representations [55,139].

At the atomic level, the experimental values based on the ionization potential and electron affinity definitions for electronegativity and chemical hardness were considered, see Equations (14) and (19), respectively, while for the polarizability new Hydrogenic quantum formulation was provided by Equation (6), and in Appendix by Equation (A22), recovering the exact value for the Hydrogen system by Equation (7), in close agreement with other available atomic quantum formulations of Equations (4) and (5). Nevertheless, the AIM level was formed by appropriate averaging of atoms-in-molecule summation for each of the considered reactivity indices, see Equations (8), (13) and (18), and along of their MOL counterparts of Equations (11), (14), and (19) the polarizability-, electronegativity- and chemical hardness- based aromaticity definitions were formulated with the associate qualitative trends established by Equations (12), (17), and (20), respectively. Yet, for MOL level of computations, all major quantum chemical methods for orbital spectra computation were considered, in Section 2.3, and implemented in the current application for some basics aromatics in Section 2. Because of the quantum observable character of polarizability the related aromaticity scale was considered as benchmark for actual study and it offered the possibility of formulating five rules for aromaticity:

*Aroma1*: the greater effect on aromaticity by mono- over bi-(poly-) benzenic rings;
*Aroma2*: the greater effect on aromaticity by CH- replaced group over H- substituted group on benzenic rings;
*Aroma3*: the greater effect on aromaticity by bi- (poly-) over mono- CH- replaced group on benzenic rings;
*Aroma4*: the greater effect on aromaticity by OH- group over NH_2_- substituted groups on benzenic rings;
*Aroma5*: the greater effect on aromaticity by the stereoisomers with substituted group having the lowest atomic charge contribution (or the lowest free valence or largest bonding order, e.g., OH- substituted group) to the benzenic rings, unless free electrons on that group exist (e.g., NH_2_- substituted group) in which case the rule is inversed.

These rules are then checked for electronegativity and chemical hardness derived-aromaticity scales with the synopsis of the results in Tables 5 and 6. It followed that chemical hardness, although generally in better agreement with these rules for most of the quantum chemical methods considered for its MOL computation, may not be considered infallible against aromaticity, at least for the reason it does not fulfils at all with the Aroms5 rule above. Surprisingly, chemical hardness index is more suited in modeling aromaticity when considered within semi-empirical computational framework, while the electronegativity responds better in conjunction with *ab initio* methods.

From quantum computational perspective, the consecrated HF method seems to get more marks in fulfillment of above Aroma1-to-5 rules, cumulated for electronegativity and chemical hardness based-aromaticity scales; it leads with the important idea the correlation effects are not determinant in aromaticity phenomenology, an idea confirmed also by the fact the density functional without exchange and correlation produces not-negligible fits with Aroma1, 2, and 4 rules in electronegativity framework.

Overall, few basic ideas in computing aromaticity should be finally emphasized

- *there is preferable computing aromaticity in an absolute manner*, *i.e.*, for each molecule based on its pre- and post- bonding properties (as is the present compactness definition, for instance) without involving other referential molecule, as is often case in the fashioned aromaticity scales;
- *the comparison between various aromaticity absolute scales is to be done respecting that one based on a structural or reactivity index with attested observational character* (as is the present polarizability based- aromaticity);
- *the rules derived from the absolute aromaticity scale based on observable quantum index should be considered for further guidance* for the rest of aromaticity scales considered;
- *the aromaticity concept*, *although currently associated with stability character of molecules*, *seems to not depending on correlation and sometimes neither by exchange effects*.

Future quests should enlarge the basis of the present conclusions by performing comparative aromaticity studies at the level of biomolecules and nanostructures; at the end of the day, the aromaticity concept in general and with its particular specialization should represent just a tool/vehicle in modeling and understanding the chemical bond of atoms in molecules and nanostructures, either in isolated or interacting states.

## Figures and Tables

**Table 1.** Main geometric and energetic characteristics for atoms involved in organic compounds considered in this work (see Table 2), as radii from Ref. [137] and polarizabilities (Pol) based upon Equation (8), along the electronegativity (χ) and chemical hardness (η) from Ref. [53,54], respectively.

**Atom**	**Radius [Å]**	**Pol [Å]^3^**	**χ [eV]**	**η [eV]**
**H**	0.529	0.666	7.18	6.45
**C**	0.49	0.529	6.24	4.99
**N**	0.41	0.310	6.97	7.59
**O**	0.35	0.193	7.59	6.14

**Table 2.** Atoms-in-Molecule (AIM) and molecular (MOL) structures, volumes, and polarizability based-aromaticities A_P_ of Equation (12), employing the atomic values of Table 1 and the ab-initio (Hartree-Fock) quantum environment computation [138]; AIM electronegativity and chemical hardness are reported (in electron-voles, eV) employing the Equations (13) and (18), respectively.

**Compound**	**Structure**			**Polarizability [Å]^3^**				**AIM-Indices**	
			
**Formula** **Name** **CAS** **Index (*πe*^−^)**	***AIM***	***Molecule***	***Conventional***	***P^AIM^***	***Molec***		**A_P_**	**χ^AIM^**	**η^AIM^**
					***Vol***	***P^MOL^***			
C_6_H_6_ **Benzene** 71-43-2 **I (6)**	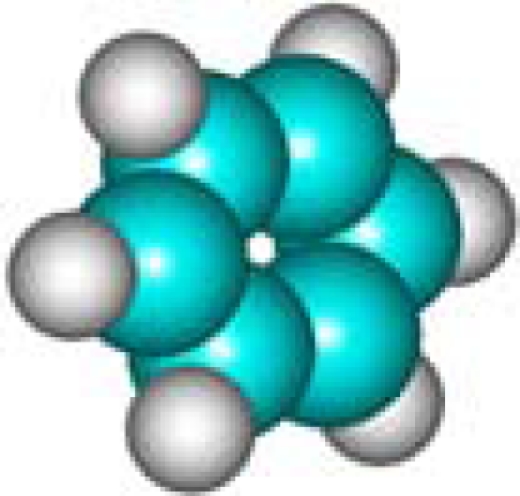	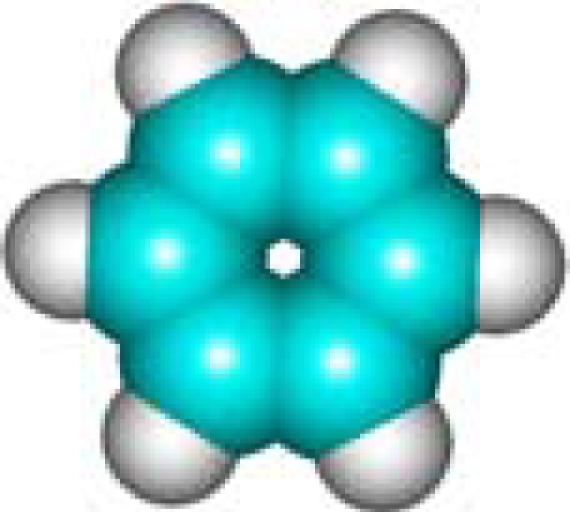	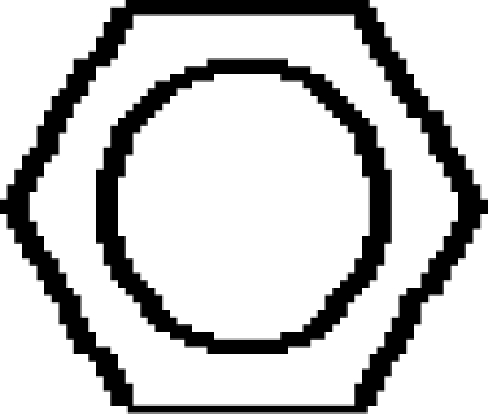	**7.17**	**328.11**	**19.58**	**0.37**	**6.68**	**5.63**

C_4_H_4_N_2_ **Pyrimidine** 289-95-2 **II (6)**	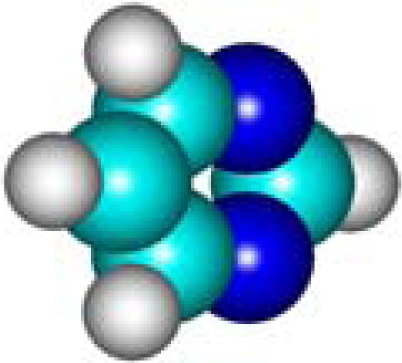	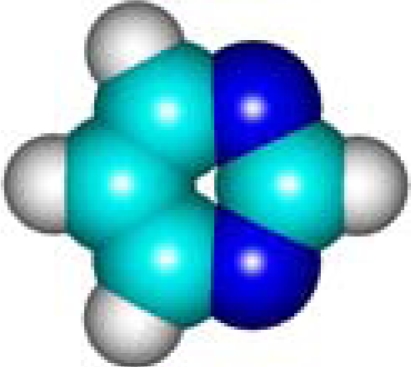	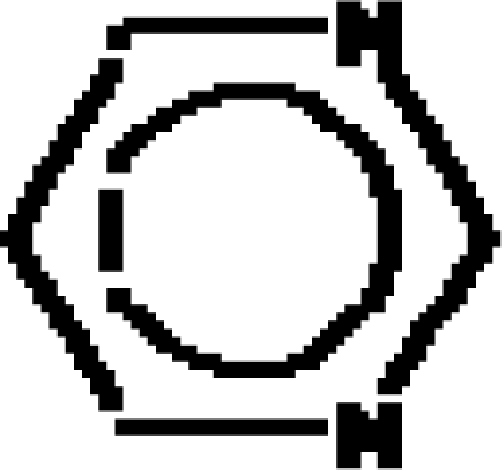	**5.40**	**306.46**	**12.19**	**0.44**	**6.73**	**5.93**

C5H5N **Pyridine** 110-86-1 **III (6)**	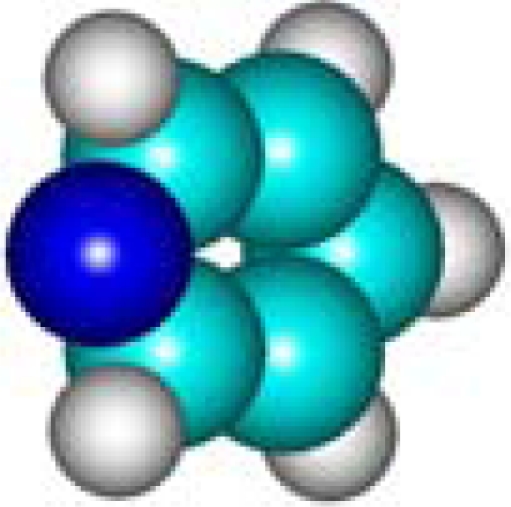	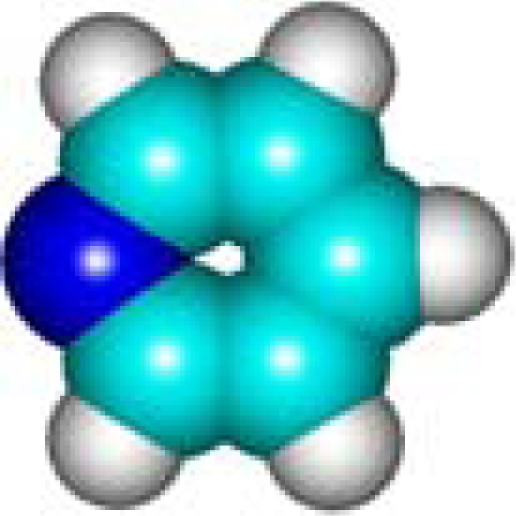	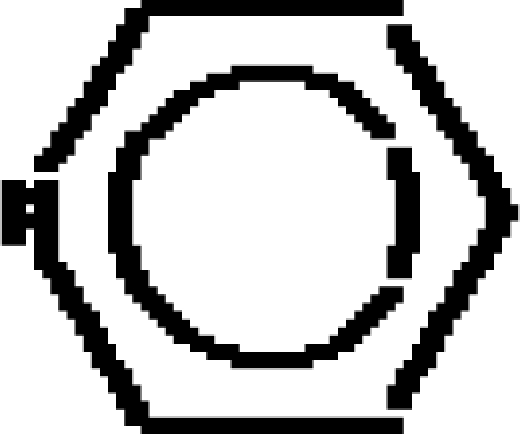	**6.29**	**320.75**	**12.76**	**0.49**	**6.70**	**5.76**

C6H6O **Phenol** 108-95-2 **IV (8)**	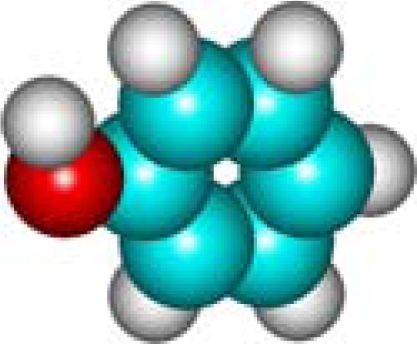	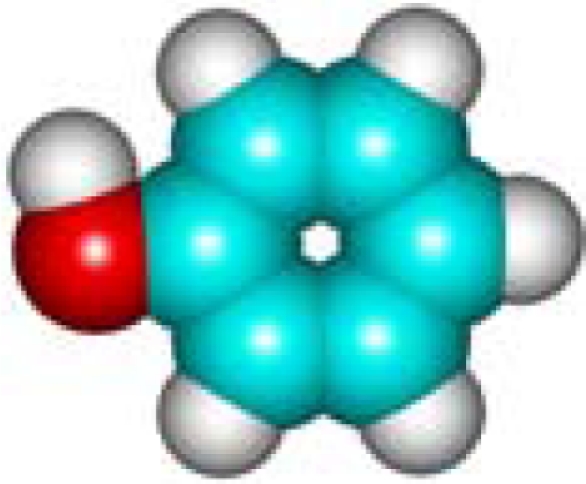	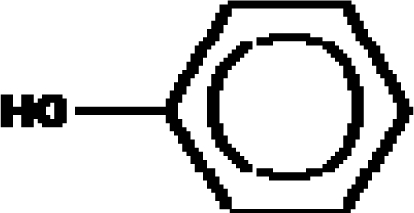	**7.37**	**356.91**	**10.65**	**0.69**	**6.74**	**5.66**

C_6_H_7_N **Aniline** 62-53-3 **V (8)**	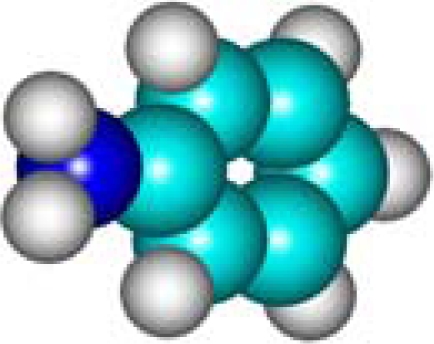	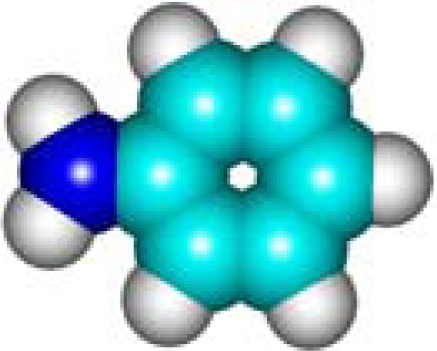	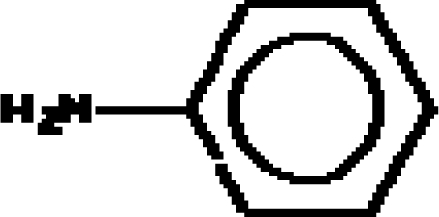	**8.15**	**371.73**	**11.09**	**0.73**	**6.73**	**5.79**

C_10_H_8_ **Naphthalene** 91-20-3 **VI (10)**	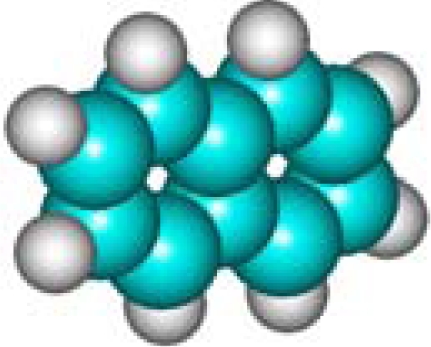	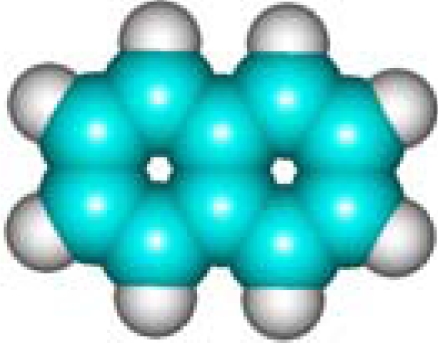	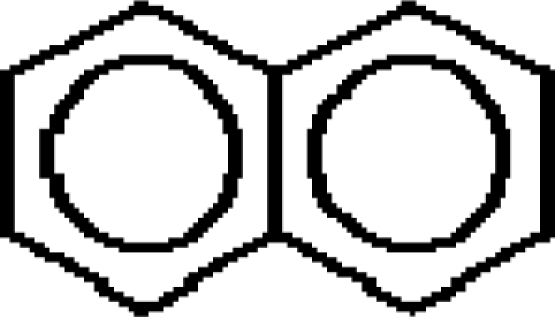	**10.62**	**463.84**	**11.07**	**0.96**	**6.63**	**5.55**

C_10_H_8_O **1-Naphthol** 90-15-3 **VII (12)**	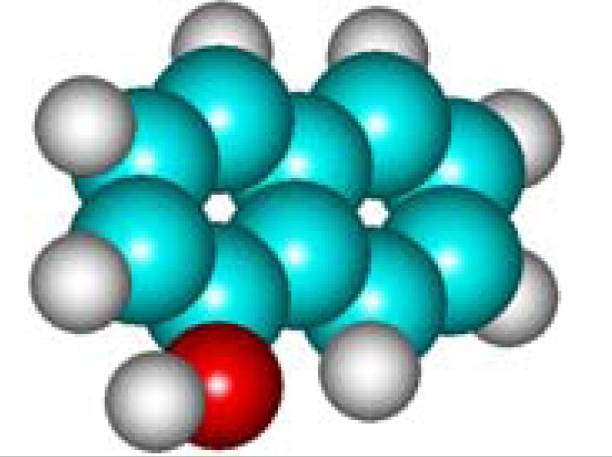	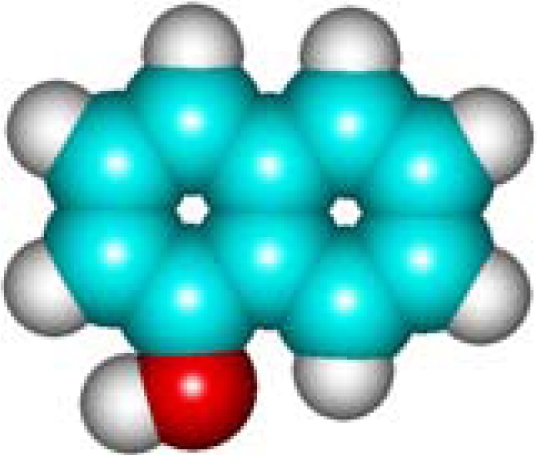	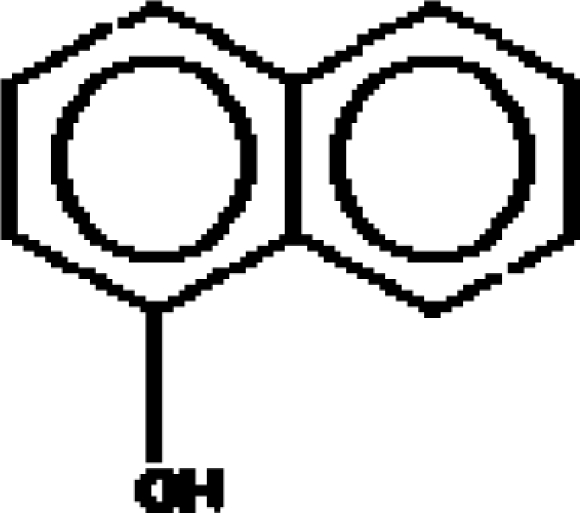	**10.82**	**483.88**	**9.63**	**1.12**	**6.67**	**5.58**

C_10_H_8_O **2-Naphthalelon** 135-19-3 **VIII (12)**	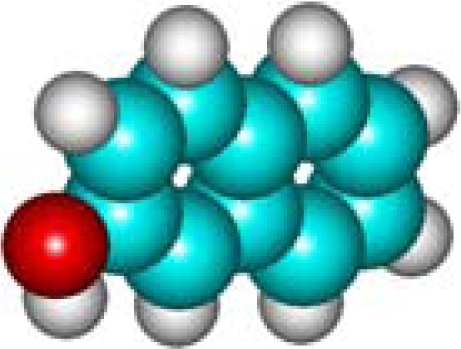	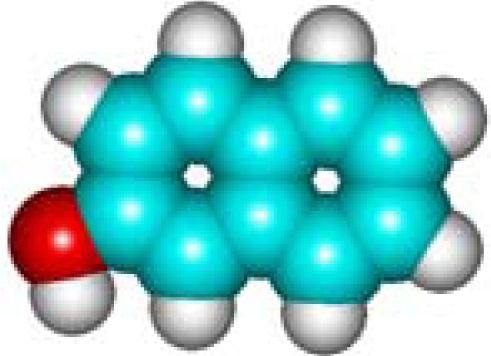	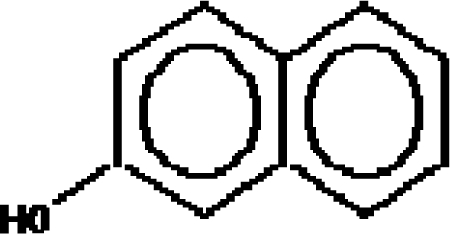	**10.82**	**478.39**	**9.52**	**1.14**	**6.67**	**5.58**

C_10_H_9_N **2-Naphthalenamine** 91-59-8 **IX (12)**	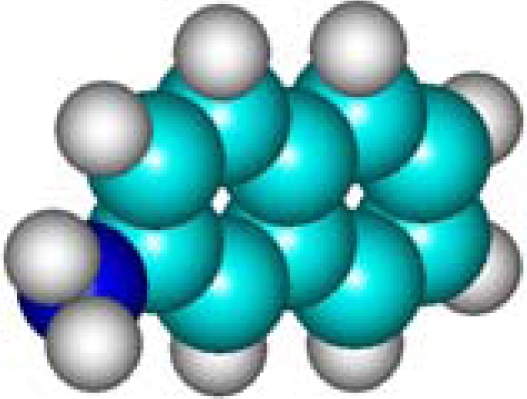	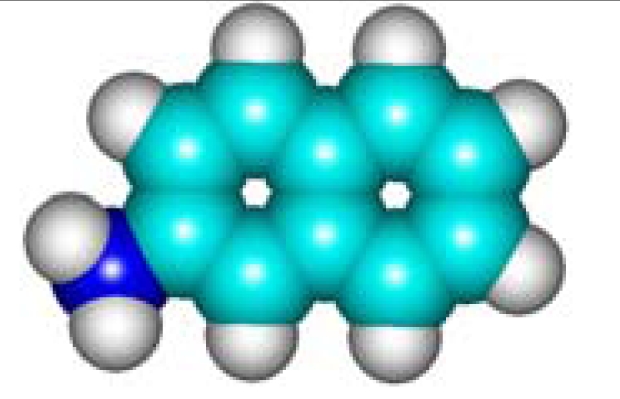	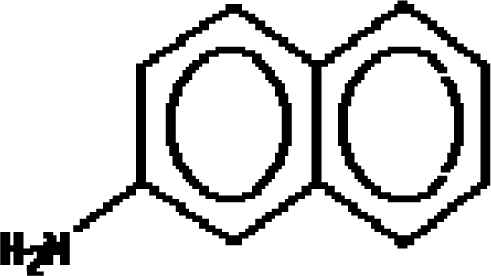	**11.60**	**501.54**	**9.98**	**1.16**	**6.67**	**5.66**

C_10_H_9_N **1-Naphthalenamine** 134-32-7 **X (12)**	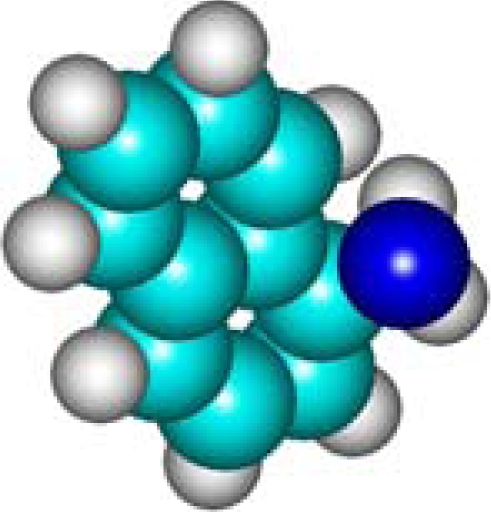	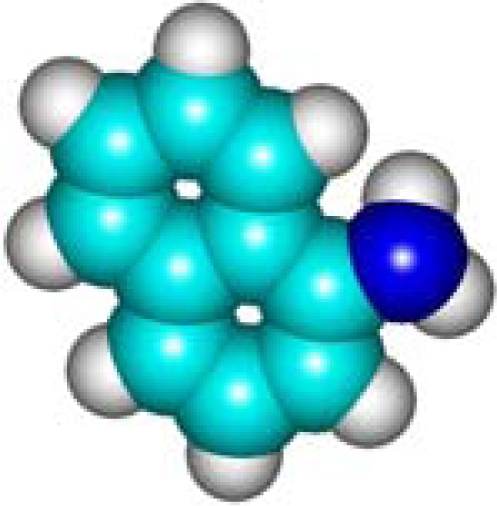	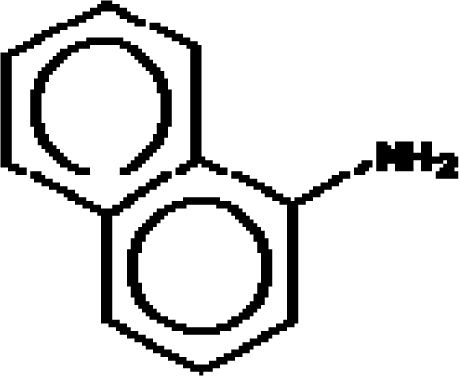	**11.60**	**496.11**	**9.87**	**1.18**	**6.67**	**5.66**

**Table 3.** Frontier HOMO and LUMO energies, the molecular electronegativity and chemical hardness of Equations (14) and (19), along the quantum compactness aromaticity A_EL_ and A_Hard_ indices for compounds of Table 2 as computed with Equations (17) and (20) within semi-empirical quantum chemical methods [138]; all energetic values in electronvolts (eV).

**Compound**		**CNDO**	**INDO**	**MINDO3**	**MNDO**	**AM1**	**PM3**	**ZINDO/1**	**ZINDO/S**
**Index**	**Property**								
**I**	E_LUMO_	3.892207	4.451804	1.26534	0.3681966	0.514791	0.3440638	7.970686	0.7950159
	–E_HOMO_	13.80296	13.24336	9.165875	9.391555	9.591248	9.652767	9.724428	8.927967
	χ	4.96	4.40	3.95	4.51	4.54	4.65	0.88	4.07
	η	8.85	8.85	5.22	4.88	5.05	4.998	8.85	4.86
	**A_EL_**	**1.35**	**1.52**	**1.69**	**1.48**	**1.472**	**1.435**	**7.62**	**1.64**
	**A_Hard_**	**0.64**	**0.64**	**1.08**	**1.15**	**1.11**	**1.13**	**0.64**	**1.16**

**II**	E_LUMO_	2.709499	3.147036	0.951945	–0.3960558	–0.2959276	–0.6894529	6.422883	–0.3419995
	–E_HOMO_	13.39755	11.86692	8.356924	10.36822	10.56194	10.62456	8.527512	9.67323
	χ	5.34	4.36	3.70	5.38	5.43	5.66	1.05	5.01
	η	8.05	7.51	4.65	4.99	5.13	4.968	7.48	4.67
	**A_EL_**	**1.26**	**1.54**	**1.82**	**1.25**	**1.24**	**1.19**	**6.40**	**1.34**
	**A_Hard_**	**0.74**	**0.79**	**1.274**	**1.189**	**1.16**	**1.19**	**0.79**	**1.271**

**III**	E_LUMO_	3.051321	3.521359	1.011715	–0.02136767	0.1085682	–0.1944273	6.909242	0.01985455
	–E_HOMO_	13.45145	12.06075	8.813591	9.692185	9.903634	10.0075	8.598721	9.040296
	χ	5.20	4.27	3.90	4.86	4.90	5.10	0.84	4.51
	η	8.25	7.79	4.91	4.84	5.01	4.91	7.75	4.53
	**A_EL_**	**1.29**	**1.57**	**1.718**	**1.38**	**1.37**	**1.31**	**7.93**	**1.49**
	**A_Hard_**	**0.6980**	**0.74**	**1.17**	**1.191**	**1.15**	**1.17**	**0.74**	**1.272**

**IV**	E_LUMO_	3.718175	4.275294	1.085692	0.1763786	0.3450922	0.2196551	7.706827	0.6566099
	–E_HOMO_	12.51092	11.71605	8.669437	9.022056	9.108171	9.169341	8.265366	8.557631
	χ	4.40	3.72	3.79	4.42	4.38	4.47	0.28	3.95
	η	8.11	8.00	4.88	4.60	4.73	4.69	7.99	4.61
	**A_EL_**	**1.53**	**1.81**	**1.78**	**1.52**	**1.538**	**1.506**	**24.13**	**1.71**
	**A_Hard_**	**0.6975**	**0.71**	**1.16**	**1.23**	**1.20**	**1.21**	**0.71**	**1.23**

**V**	E_LUMO_	4.002921	4.61612	1.360785	0.5461559	0.7090454	0.5768315	8.106442	0.8517742
	–E_HOMO_	11.22051	10.28413	7.783539	8.207099	8.186989	8.028173	6.803807	7.95583
	χ	3.61	2.83	3.21	3.83	3.74	3.73	–0.65	3.55
	η	7.61	7.45	4.57	4.38	4.45	4.30	7.46	4.40
	**A_EL_**	**1.86**	**2.37**	**2.10**	**1.76**	**1.80**	**1.806**	**–10.33**	**1.89**
	**A_Hard_**	**0.76**	**0.78**	**1.266**	**1.323**	**1.3017**	**1.35**	**0.78**	**1.31**

**VI**	E_LUMO_	2.172528	2.757336	0.4589255	–0.3423392	–0.2855803	–0.4525464	6.197386	–0.04161556
	–E_HOMO_	11.48051	10.89619	8.165956	8.544642	8.660414	8.746719	7.4545	7.835637
	χ	4.65	4.07	3.85	4.44	4.47	4.60	0.63	3.939
	η	6.83	6.83	4.31	4.10	4.19	4.15	6.83	3.90
	**A_EL_**	**1.42**	**1.63**	**1.721**	**1.49**	**1.48**	**1.441**	**10.55**	**1.68**
	**A_Hard_**	**0.81**	**0.81**	**1.29**	**1.35**	**1.33**	**1.34**	**0.81**	**1.42**

**VII**	E_LUMO_	2.192621	2.79537	0.5106197	–0.3850094	–0.2975906	–0.4355633	6.210848	–0.06489899
	–E_HOMO_	10.95387	10.26489	7.918682	8.376475	8.441528	8.514781	6.859143	7.681855
	χ	4.38	3.73	3.70	4.38	4.37	4.48	0.32	3.87
	η	6.57	6.53	4.21	4.00	4.07	4.04	6.54	3.81
	**A_EL_**	**1.52**	**1.79**	**1.80**	**1.52**	**1.53**	**1.49**	**20.58**	**1.72**
	**A_Hard_**	**0.85**	**0.85**	**1.32**	**1.40**	**1.37**	**1.38**	**0.85**	**1.47**

**VIII**	E_LUMO_	2.534854	3.128462	0.5805296	–0.3075339	–0.2500397	–0.3581562	6.510067	0.08197734
	–E_HOMO_	11.50232	10.80223	8.246805	8.747499	8.821697	8.887013	7.405916	7.956836
	χ	4.48	3.84	3.83	4.53	4.54	4.62	0.45	3.937
	η	7.02	6.97	4.41	4.22	4.29	4.26	6.96	4.02
	**A_EL_**	**1.49**	**1.74**	**1.74**	**1.47**	**1.471**	**1.443**	**14.89**	**1.69**
	**A_Hard_**	**0.795**	**0.80**	**1.26**	**1.322**	**1.302**	**1.31**	**0.80**	**1.39**

**IX**	E_LUMO_	2.228869	2.844	0.5107521	–0.3597778	–0.2714103	–0.4318241	6.286494	–0.01188275
	–E_HOMO_	10.94335	10.11959	7.783869	8.371226	8.367208	8.374782	6.666291	7.655258
	χ	4.36	3.64	3.64	4.37	4.32	4.40	0.19	3.83
	η	6.59	6.48	4.15	4.01	4.05	3.97	6.48	3.82
	**A_EL_**	**1.53**	**1.83**	**1.83**	**1.53**	**1.544**	**1.515**	**35.12**	**1.74**
	**A_Hard_**	**0.86**	**0.87**	**1.36**	**1.41**	**1.40**	**1.43**	**0.87**	**1.48**

**X**	E_LUMO_	2.22685	2.840066	0.5115107	–0.3805175	–0.2805806	–0.4578161	6.326563	–0.00408988
	–E_HOMO_	10.68815	9.865701	7.676749	8.272097	8.261106	8.2799	6.414326	7.540981
	χ	4.23	3.51	3.58	4.33	4.27	4.37	0.04	3.77
	η	6.46	6.35	4.09	3.95	3.99	3.91	6.37	3.77
	**A_EL_**	**1.58**	**1.90**	**1.86**	**1.54**	**1.56**	**1.53**	**152.0**	**1.77**
	**A_Hard_**	**0.88**	**0.89**	**1.38**	**1.43**	**1.42**	**1.45**	**0.89**	**1.50**

**Table 4.** The same quantities of Table 3 as computed within various *ab initio* approaches: by Density Functional Theory without exchange-correlation (noEX-C), and with B3-LYP, B3-PW91, and Becke97 exchange-correlations, and by Hartree-Fock method, all with minimal (STO-3G) basis sets.

**Compound**		**DFT**					**Hartree-Fock**

**Index**	**Property**	**noEX-C**	**B3-LYP**	**B3-PW91**	**EDF1**	**Becke97**	
**I**	E_LUMO_	15.69352	2.52946	2.398649	1.561805	2.512676	7.234344
	–E_HOMO_	–8.870216	5.158205	5.338667	4.430191	5.165561	7.502962
	χ	–12.28	1.31	1.47	1.43	1.33	0.13
	η	3.41	3.84	3.87	3.00	3.84	7.37
	**A_EL_**	**–0.54**	**5.08**	**4.54**	**4.66**	**5.04**	**49.74**
	**A_Hard_**	**1.65**	**1.46**	**1.46**	**1.88**	**1.47**	**0.76**

**II**	E_LUMO_	15.11303	0.9238634	0.7736028	–0.04114805	0.9030221	5.579984
	–E_HOMO_	–13.04602	4.744987	4.883547	3.513406	4.728943	8.695125
	χ	–14.08	1.91	2.05	1.78	1.91	1.56
	η	1.03	2.83	2.83	1.74	2.82	7.14
	**A_EL_**	**–0.478**	**3.52**	**3.27**	**3.79**	**3.52**	**4.32**
	**A_Hard_**	**5.74**	**2.09**	**2.096**	**3.42**	**2.106**	**0.83**

**III**	E_LUMO_	15.34953	1.622094	1.477663	0.6587179	1.60312	6.284506
	–E_HOMO_	–12.73475	4.751619	4.893573	3.484843	4.739381	7.943096
	χ	–14.04	1.56	1.71	1.41	1.57	0.83
	η	1.31	3.19	3.186	2.07	3.1713	7.11
	**A_EL_**	**–0.477**	**4.28**	**3.92**	**4.74**	**4.27**	**8.08**
	**A_Hard_**	**4.41**	**1.81**	**1.81**	**2.78**	**1.82**	**0.81**

**IV**	E_LUMO_	16.5171	2.596515	2.475588	1.716375	2.584044	7.102361
	–E_HOMO_	–12.45941	3.760901	3.909865	2.872823	3.758234	6.672404
	χ	–14.49	0.58	0.72	0.58	0.59	–0.21
	η	2.03	3.18	3.193	2.29	3.1711	6.89
	**A_EL_**	**–0.465**	**11.58**	**9.40**	**11.66**	**11.48**	**–31.35**
	**A_Hard_**	**2.79**	**1.78**	**1.77**	**2.47**	**1.78**	**0.82**

**V**	E_LUMO_	16.5102	2.963498	2.848121	2.077958	2.949314	7.449772
	–E_HOMO_	–12.06327	3.094653	3.234635	2.291472	3.087551	5.765693
	χ	–14.29	0.07	0.19	0.11	0.07	–0.84
	η	2.22	3.03	3.04	2.18	3.02	6.61
	**A_EL_**	**–0.471**	**102.63**	**34.82**	**63.04**	**97.37**	**–7.99**
	**A_Hard_**	**2.60**	**1.91**	**1.90**	**2.65**	**1.92**	**0.88**

**VI**	E_LUMO_	14.5038	1.290144	1.146572	0.4413206	1.267581	5.544161
	–E_HOMO_	–10.0267	4.156837	4.331527	3.541704	4.159986	6.084805
	χ	–12.27	1.43	1.59	1.55	1.45	0.27
	η	2.24	2.72	2.74	1.99	2.71	5.81
	**A_EL_**	**–0.54**	**4.63**	**4.16**	**4.28**	**4.58**	**24.53**
	**A_Hard_**	**2.48**	**2.04**	**2.03**	**2.79**	**2.05**	**0.95**

**VII**	E_LUMO_	15.40361	1.534507	1.39925	0.7539564	1.51676	5.631796
	–E_HOMO_	–12.32641	3.422596	3.578508	2.691508	3.420128	5.689867
	χ	–13.87	0.94	1.09	0.97	0.95	0.03
	η	1.54	2.48	2.49	1.72	2.47	5.66
	**A_EL_**	**–0.481**	**7.07**	**6.12**	**6.88**	**7.01**	**229.72**
	**A_Hard_**	**3.63**	**2.25**	**2.24**	**3.24**	**2.26**	**0.99**

**VIII**	E_LUMO_	15.48911	1.614079	1.472582	0.8028092	1.593253	5.815819
	–E_HOMO_	–12.3533	3.699537	3.860561	2.910033	3.698387	6.201466
	χ	–13.92	1.04	1.19	1.05	1.05	0.19
	η	1.57	2.66	2.67	1.86	2.65	6.01
	**A_EL_**	**–0.479**	**6.40**	**5.59**	**6.33**	**6.34**	**34.59**
	**A_Hard_**	**3.56**	**2.10**	**2.093**	**3.01**	**2.109**	**0.93**

**IX**	E_LUMO_	15.08743	1.524559	1.389197	0.7397588	1.502934	5.626748
	–E_HOMO_	–11.85368	3.370883	3.52209	2.624703	3.369097	5.680253
	χ	–13.47	0.92	1.07	0.94	0.93	0.03
	η	1.62	2.45	2.46	1.68	2.44	5.65
	**A_EL_**	**–0.495**	**7.23**	**6.25**	**7.08**	**7.15**	**249.32**
	**A_Hard_**	**3.50**	**2.31**	**2.30**	**3.36**	**2.32**	**1.00**

**X**	E_LUMO_	15.12512	1.53895	1.404091	0.7564005	1.518818	5.640772
	–E_HOMO_	–11.90494	3.264767	3.41559	2.541048	3.262239	5.520739
	χ	–13.52	0.86	1.01	0.89	0.87	–0.06
	η	1.61	2.40	2.41	1.65	2.39	5.58
	**A_EL_**	**–0.494**	**7.73**	**6.63**	**7.47**	**7.65**	**–111.14**
	**A_Hard_**	**3.52**	**2.36**	**2.35**	**3.43**	**2.37**	**1.01**

**Table 5.** The fulfillment (×) of the aromaticity (Aroma1–5) rules abstracted from polarizability based scale in the case of *electronegativity* based-aromaticity records of Tables 3 and 4 for the molecules of Table 2.

** Aromaticity Rules** **Quantum Methods**		Aroma1	Aroma2	Aroma3	Aroma4	Aroma5
**Semi-empirical**	*CNDO*	−	−	−	−	−
	*INDO*	−	−	−	−	−
	*MINDO3*	−	−	×	−	−
	*MNDO*	−	−	−	−	−
	*AM1*	−	−	−	−	−
	*PM3*	−	−	−	−	−
	*ZINDO/1*	−	−	−	−	−
	*ZINDO/S*	−	−	−	−	−

**Ab initio**	*noEXc*	×	×	−	×	−
	*B3-LYP*	×	−	−	−	−
	*B3-PW91*	×	−	−	−	−
	*EDF1*	×	−	−	−	−
	*Becke97*	×	−	−	−	−
	*Hartree-Fock*	−	×	−	−	×

**Table 6.** The same check for the present aromaticity rules as in Table 5–yet here for the *chemical hardness* based-aromaticity scale.

** Aromaticity Rules** **Quantum Methods**		Aroma1	Aroma2	Aroma3	Aroma4	Aroma5
**Semi-empirical**	*CNDO*	×	−	−	×	−
	*INDO*	×	−	−	×	−
	*MINDO3*	×	−	−	×	−
	*MNDO*	×	−	×	×	−
	*AM1*	×	×	−	×	−
	*PM3*	×	×	−	×	−
	*ZINDO/1*	×	−	−	×	−
	*ZINDO/S*	×	−	×	×	−

**Ab initio**	*noEXc*	−	−	−	−	−
	*B3-LYP*	×	−	−	×	−
	*B3-PW91*	×	−	−	×	−
	*EDF1*	−	−	−	×	−
	*Becke97*	×	−	−	×	−
	*Hartree-Fock*	×	×	−	×	−

## Appendix: New Hydrogenic Polarizability Formula

Starting from the consecrated second order perturbation energy [80]:

(A1) $$E^{\left(2\right)}=\sum_{k\neq n}\frac{{\left|〈n|H_{1}|k〉\right|}^{2}}{E_{k}-E_{n}}$$

is specialized for the Stark potential produced by the applied external electric field with the amplitude *ɛ* in the 0*x* direction:

(A2) $$H_{1}=V(x)=-{xZe}_{0}ɛ$$

under the form:

(A3) $$E^{\left(2\right)}=-\frac{1}{2}{\alpha ɛ}^{2}$$

that allows for *α*– polarizability in (A3) the general hydrogenoid (*Z*-dependent) formulation

(A4) $$\alpha ={2e}_{0}^{2}Z^{2}\sum_{k\neq n}\frac{{\left|〈n|x|k〉\right|}^{2}}{E_{n}-E_{k}}$$

where

(A5) $$e_{0}^{2}=\frac{e^{2}}{{4\pi ɛ}_{0}}$$

is the reduced squared elementary charge

Now, to evaluate the atomic polarizability in terms of the quantum basic information contained within the atomic quantum numbers (e.g., *n*, *k*), one starts recognizing the general operatorial identity over the complete set of quantum (eigen) states:

(A6) $$\sum_{k}{\left|〈n\left|\hat{O}\right|k〉\right|}^{2}=\sum_{k}〈n\left|\hat{O}\right|k〉〈k\left|\hat{O}\right|n〉=〈n|\hat{O}\underset{1}{\underset{︸}{\left\{\sum_{k}\left|k〉〈k\right|\right\}}}\hat{O}|n〉=〈n\left|{\hat{O}}^{2}\right|n〉$$

Equation (6) is eventually known as the sum rule of Bethe and Jackiw [140,141], while its simplest dipole matrix element sum rule casts as:

(A7) $$\sum_{k}{\left|〈n|x|k〉\right|}^{2}=〈n|x^{2}|n〉$$

On the other hand, recalling the basic quantum commutation rule of momentum with space coordinate:

(A8) $$[p,\;x]=\frac{\hbar }{i}$$

along the companion energy-coordinate commutator:

(A9) $$[H,\;x]=\left[\frac{p^{2}}{2m}+V(x),x\right]=\frac{1}{2m}\left[p^{2},x\right]=\frac{\hbar }{mi}p$$

there can be inferred the quantum relationship:

(A10) $$\frac{\hbar }{i}=〈n|(px-xp)|n〉=\sum_{k}\left\{〈n|p|k〉〈k|x|n〉-〈n|x|k〉〈k|p|n〉\right\}$$

upon inserting of the above quantum closure relation over the complete set of eigen-states. The first term in the right-hand side of the last expression may be reformulated as:

(A11) $$〈n|p|k〉=〈n|\frac{mi}{\hbar }[H,x]|k〉=\frac{mi}{\hbar }〈n|(Hx-xH)|k〉=\frac{mi}{\hbar }(E_{n}-E_{k})〈n|x|k〉$$

and along the similar relation that springs out from the second term in (A10) one gets the equation:

(A12) $$\frac{\hbar }{i}=\frac{mi}{\hbar }\sum_{k}[(E_{n}-E_{k})-(E_{k}-E_{n})]{\left|〈n|x|k〉\right|}^{2}$$

that can be rearranged under the so called Thomas-Reiche-Kuhn (TRK) energy-weighted sum rule [142–144]:

(A13) $$\frac{\hbar^{2}}{2m}=\sum_{k}\left(E_{k}-E_{n}\right){\left|〈n|x|k〉\right|}^{2}$$

Remarkably, the expansion (A13) may be also obtained by requiring that the Kramers-Heisenberg dispersion relation reduce to the Thomas scattering formula at high energies; indeed, through rewriting Equation (A13) in the form:

(A14) $$\sum_{k}\frac{2m(E_{k}-E_{n})}{\hbar^{2}}{\left|〈n|x|k〉\right|}^{2}=\sum_{k}f_{n,k}=1$$

it provides an important theoretical support for the experimental checks of the oscillator strengths (*f_n,k_*) as a confirmation of early quantum results [145,146].

Now, returning to the evaluation of polarizability given by (A4) one can use the recipe (A13) to facilitate the skipping out of the energy-singularity towards the all-eigen-state summation (A7) with the successive results:

(A15) $$\begin{array}{l} \alpha ={2e}_{0}^{2}Z^{2}\sum_{k\neq n}\frac{{\left|〈n|x|k〉\right|}^{2}}{E_{n}-E_{k}}=2\frac{{2me}_{0}^{2}}{\hbar^{2}}Z^{2}\frac{\hbar^{2}}{2m}\sum_{k\neq n}\frac{{\left|〈n|x|k〉\right|}^{2}}{E_{n}-E_{k}}=\frac{{4me}_{0}^{2}}{\hbar^{2}}Z^{2}\left\{\sum_{k}\left(E_{k}-E_{n}\right){\left|〈n|x|k〉\right|}^{2}\right\}\sum_{k\neq n}\frac{{\left|〈n|x|k〉\right|}^{2}}{E_{n}-E_{k}}\\ \overset{\text{all}\;k}{\to }2\frac{{4me}_{0}^{2}}{\hbar^{2}}Z^{2}{\left(\sum_{k}{\left|〈n|x|k〉\right|}^{2}\right)}^{2}=8\frac{{me}_{0}^{2}}{\hbar^{2}}Z^{2}{\left|〈n|x^{2}|n〉\right|}^{2}=8\frac{Z^{2}}{a_{0}}{\left|〈n|x^{2}|n〉\right|}^{2} \end{array}$$

where we recognized the first Bohr radius expression:

(A16) $$a_{0}=\frac{\hbar^{2}}{e_{0}^{2}m}$$

Finally, the obtained expression (A15) is unfolded through replacing the coordinate observation with the atomic radius quantum average displacement respecting its instantaneous value:

(A17) $$x\to r-{〈r〉}_{nl}$$

It allows the immediate formation of the squared coordinate expression:

(A18) $$x^{2}=r^{2}-2r{〈r〉}_{nl}+{〈r〉}_{nl}^{2}$$

of which the observed quantum average looks like

(A19) $$〈n\left|x^{2}\right|n〉\to {〈x^{2}〉}_{nl}={〈r^{2}〉}_{nl}-{〈r〉}_{nl}^{2}$$

The replacement of Equation (A19) in the polarizability (A15) produces its radial averages’ dependency:

(A20) $$\alpha =8\frac{Z^{2}}{a_{0}}{\left[{〈r^{2}〉}_{nl}-{〈r〉}_{nl}^{2}\right]}^{2}$$

Knowing the first and second order quantum averages for the atomic radius of a Hydrogenic system written in terms of the principal and azimuthal quantum numbers *n* and *l*, respectively [147]:

(A21a) $${〈r〉}_{nl}=\frac{1}{2}\left(\frac{a_{0}}{Z}\right)\left[{3n}^{2}-l(l+1)\right]$$

(A21b) $${〈r^{2}〉}_{nl}=\frac{1}{2}{\left(\frac{a_{0}}{Z}\right)}^{2}n^{2}\left[{5n}^{2}-3l(l+1)+1\right]$$

the static atomic polarizability (A20) takes the analytical form:

(A22) $$\alpha_{nl}(Z)=\frac{a_{0}^{3}}{{2Z}^{2}}{\left[n^{2}\left(2+n^{2}\right)-l^{2}{\left(1+l\right)}^{2}\right]}^{2}$$

recovering the exact result for the Hydrogen limiting case:

(A23) $$\alpha_{n=1,l=0}(Z=1)=\frac{9}{2}a_{0}^{3}$$

It is worth noting that the present derivation relays on the second order perturbation energy (A1) while the final expression (A22) is assumed to be exact through the Hydrogen checking case (A23), although different by the other reported also as valid formulations, see Equations (4) and (5) in the main text and References [45–47]. Nevertheless, the present atomic polarizability, either under expressions (A15) or (A22) is to be further tested for reliability in modeling of atomic (or ionic) and molecular systems.
